# Rational Design of Single-Phase High-Entropy Oxides via Large Language Model Data Mining and Explainable Machine Learning

**DOI:** 10.1021/acs.jcim.6c00752

**Published:** 2026-04-25

**Authors:** Arthur da Silva Sousa Santos, Elena Stojanovska, Antonio Augusto Alves, Amauri Jardim de Paula, Daniel Zanetti de Florio, James Moraes de Almeida

**Affiliations:** † Center for Engineering, Modeling and Applied Social Sciences, 74362 Federal University of ABC (UFABC), Av. dos Estados, 5001, Bangú, Santo André, São Paulo 09210-580, Brazil; ‡ Independent Researcher, Trento, TN 38122, Italy; § Ilum School of Science, Brazilian Center for Research in Energy and Materials (CNPEM), Rua Lauro Vannucci, 1020, Fazenda Santa Cândida, Campinas, São Paulo 13083-970, Brazil; ∥ 74360 Aeronautics Institute of Technology (ITA), Praça Marechal Eduardo Gomes, 50, Vila das Acacias, São José dos Campos, São Paulo 12228-900, Brazil; ⊥ Center of Natural and Human Sciences, Federal University of ABC (UFABC), Av. dos Estados, 5001, Bangú, Santo André, São Paulo 09210-580, Brazil

## Abstract

The rational design of high-entropy oxides (HEOs) is currently hindered by the scarcity of structured property data in the scientific literature. In this work, we present an end-to-end materials informatics framework that couples large language model (LLM) data mining with interpretable machine learning to predict single-phase stability in HEOs. We deployed agents based on gpt-oss-120b to extract compositions, phases, and synthesis methods from unstructured scientific abstracts. Combined with regular-expression routines, the LLM-based agent achieved an accuracy of 96% in database generation despite the complexity of the task, including on-the-fly inference of relative cation proportions. Subsequently, a range of machine-learning models was trained in an exploratory multiclass classification setting to distinguish canonical HEO crystal structures using several variants of the primary databases obtained by combining different feature subsets. For this task, an XGBoost classifier achieved an F_1_-score of 86% in a seven-class classification problem, and the best-performing database variant combined primary and statistical features. This optimal database representation was then used to train a neural-network binary classifier to distinguish perovskite from nonperovskite compositions, achieving 97.9% classification accuracy on the test set, whereas the Goldschmidt tolerance factor reached only 67.3% on the same data. These results indicate that the proposed methodology can support the design of HEO compositions with target properties and substantially outperforms traditional descriptor-based approaches. Furthermore, SHAP (SHapley Additive exPlanations) analysis revealed that high-entropy perovskite phase stability is governed by a critical interplay between geometric factors, such as the sum of cation radii, and electronic descriptors, including Sanderson electronegativity and atomization enthalpy. Overall, these findings demonstrate that LLM-driven data mining can overcome data bottlenecks and enable the discovery of physical design rules for complex, multicomponent ceramics.

## Graphical abstract

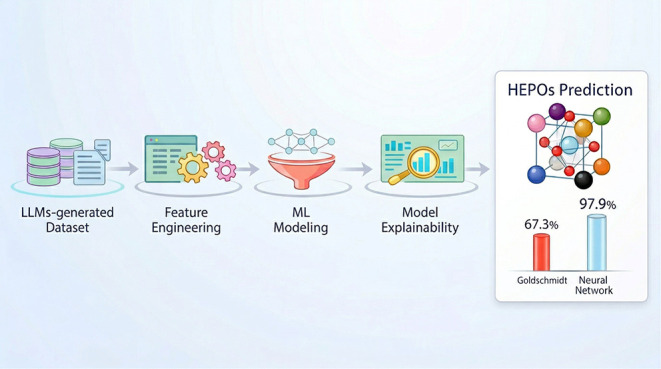

## Introduction

The concept of high-entropy materials was first introduced in the context of high-entropy alloys (HEAs) by Yeh et al.^1^ Subsequently, this idea was extended to ceramics in the seminal work of Rost et al.,^2^ who synthesized the first bulk high-entropy oxide (HEO). Since then, the distinctive characteristics of this class of materials have attracted increasing attention, as high-entropy-related effects can be exploited to achieve combinations of high thermal, chemical, and mechanical stability together with diverse functional properties.^3,4^

High-entropy oxides are ionic ceramics in which oxygen occupies the anion sites, whereas the cation sublattice is randomly populated by a mixture of five or more distinct cations. More specifically, a material is typically classified as a high-entropy oxide when its configurational entropy exceeds 1.5*R*, where *R* is the ideal gas constant.^5^ Eq 1 shows how the configurational entropy is calculated, emphasizing that it depends only on the number and relative fractions of unique atomic species that substitute a given crystallographic site.^6^

1 $$S_{\mathrm{config}}=-R\left[{\left(\sum_{i=1}^{N}x_{i}\;\ln x_{i}\right)}_{\mathrm{cation}-\mathrm{site}}+{\left(\sum_{j=1}^{M}x_{j}\;\ln x_{j}\right)}_{\mathrm{anion}-\mathrm{site}}\right]$$

where *N* and *M* are the numbers of species and *x*_*i*,*j*_ is the concentration of species *i*,*j*.

High entropy is commonly associated with four characteristic effects: entropic stabilization, lattice distortion, sluggish diffusion, and cocktail effect.^5^ These effects are particularly attractive for meeting current demands in energy storage and conversion technologies, which are rapidly expanding in response to global efforts to reduce greenhouse gas emissions; the energy sector alone accounts for approximately 75% of global greenhouse gas emissions.^7^ In catalysis, for example, HEOs enable the realization of combinations of properties that are otherwise difficult to achieve simultaneously, such as high chemical reactivity together with excellent stability.^6,8^

Although the vast compositional space of HEOs is highly attractive from a property-optimization perspective, it also poses a major challenge for materials design.^4,9^ Currently, only a limited number of studies focus on the systematic design of HEOs, and most synthesis efforts still rely on trial-and-error strategies.^10^ Furthermore, due to the presence of long-range order and the resulting large configurational space, *ab initio* simulations quickly become prohibitively expensive for the efficient exploration and design of HEOs.^9^ In this scenario, machine learning emerges as a promising route for the rational design of HEOs.^9^

However, because HEO data remain scarce in standard materials databases,^9^ the application of machine learning to HEO design still depends on strategies capable of overcoming this bottleneck. Kim et al.^11^ experimentally constructed a data set comprising 34 compositional variants of a series of high-entropy perovskites and applied machine learning to identify an optimal high-entropy perovskite composition for SOFC electrodes, ultimately proposing Ba_0.9_Cs_0.1_(Ca_0.2_Gd_0.2_La_0.2_Pr_0.2_Sr_0.2_)­Co_1.5_Fe_0.5_O_6_ (CsBaHEO) as a universal air/steam electrode. Ma et al.^12^ manually carried out an exhaustive literature survey to gather a likewise small but labor-intensive curated data set containing 66 samples, which was then used to train machine-learning models to predict high-entropy spinel phases, achieving 88% accuracy in phase classification. Duan et al.^13^ experimentally synthesized and characterized 381 oxide compositions, spanning unary to quinary cation combinations, to construct a data set for machine-learning studies of high-entropy oxides for catalytic oxidation; their models achieved 70% accuracy in predicting spinel compositions with high catalytic activity toward CH_4_ oxidation. Mints et al.^14^ generated an experimental data set within a restricted compositional space to train models for predicting OER catalytic activity, obtaining *R* ^2^ values between 0.58 and 0.64. Mehrabi-Kalajahi et al.^15^ manually extracted data from eight literature sources and combined them with newly generated data to build a data set for training an XGBoost model to predict selectivity in oxidation reactions of HEOs, achieving an error below 2.5%. Liu et al.^16^ used data compiled in a single review paper together with ADASYN techniques to train models that classify HEO compositions as fluorite, rock salt, or spinel, reaching accuracies close to 98%. Taken together, these studies highlight both the promise of data-driven HEO design and a persistent limitation: progress still relies heavily on small, manually curated, or experimentally costly data sets. In this context, recent advances in artificial intelligence, particularly large language models (LLMs), offer a new opportunity to accelerate data set construction from the scientific literature and to uncover patterns that would be difficult to extract through manual analysis alone.^17^

According to Zimmermann et al.,^18^ LLM applications across the scientific research lifecycle can be grouped into seven broad categories, including knowledge extraction and reasoning, hypothesis generation and evaluation, research data management, property prediction, molecular and materials design, automation and novel interfaces, and communication and education. Within this broader landscape, the present study is best situated within the category of knowledge extraction and reasoning, as it uses LLMs not only to extract structured information from the literature but also to interpret, organize, and transform this information into a data set suitable for subsequent machine-learning modeling.

More broadly, the use of LLMs to mine latent knowledge from scientific texts, convert that knowledge into structured data sets, and subsequently train machine-learning models has begun to emerge as a promising paradigm across different materials-related problems. Itani et al.,^19^ for example, used GPT-4o to construct an original data set of magnetic materials and subsequently trained a Random Forest classifier that achieved 90% accuracy in distinguishing ferromagnetic (FM), antiferromagnetic (AFM), and nonmagnetic (NM) materials. Lee et al.^20^ employed GPT-3.5 ^21^ to extract experimental oxidation-potential data from 74 scientific articles, assembling a data set of 592 organic molecules; using this mined data set, they trained eXtreme Gradient Boosting and Kernel Ridge Regression models that achieved prediction errors on the order of the experimental uncertainty (0.2 V). Likewise, Zhou et al.^22^ used a series of LLMs, including Llama,^23^ Qwen,^24^ and DeepSeek,^25^ to systematically extract and structure compositional and performance data from dental ceramics research articles, after which an Extra Trees model achieved an F_1_-score of 92.8% in the classification of flexural strength. These studies collectively indicate that the LLM → data set → machine learning workflow is emerging as a promising strategy for scientific discovery in data-scarce domains.

Despite the rapid progress of natural language processing in materials science, most previous studies have concentrated predominantly on named entity recognition (NER) tasks.^26^ By contrast, the present work is more appropriately framed within the still challenging paradigm of relation extraction (RE),^26^ because the LLM-based agents were tasked not only with identifying entities such as chemical compositions, but also with correctly associating each reported composition with its corresponding crystal structure and synthesis method. Using the labels reported for the chemical formula and the observed phase, the LLM agents further had to identify the crystallographic sites involved and then correctly compute the proportion of each atom in the overall composition by accounting for substitutions across the available crystallographic sites. The satisfactory execution of these tasks using LLMs is in itself a promising result, since such a workflow would be difficult to implement using explicit rule-based programming alone and, at the same time, prohibitively laborious if performed manually across thousands of texts. It also places the present study within the still underexplored field of LLMs for quantitative chemistry, understood here as tasks requiring explicit computational reasoning, typically involving the application of formulas, step-by-step calculations, and numerical outputs such as floating-point values.^27^

Although LLMs have already demonstrated considerable potential in materials science,^17,18^ they have not yet been applied to the high-entropy oxide phase-formation problem. In the present work, we employ the large language model *gpt-oss-120b* ^28^ to programmatically extract data from the scientific literature, thereby constructing original databases of HEO compositions, crystal phases, and synthesis methods. Phase prediction is formulated as a supervised classification problem in which the target variable is the crystal structure, and the descriptors are features computed from composition.

First, the agents responsible for constructing the primary database were evaluated against a manually curated benchmark, resulting in a high data-generation accuracy of 96%. The largest primary database obtained comprises 711 high-entropy oxide samples with their corresponding crystal phases. Next, we carried out an exploratory stage in which multiple variants of this database, differing in the types of feature sets employed, were used to train a large number of shallow machine-learning models for a seven-class crystal-structure classification task (spinel, perovskite, fluorite, pyrochlore, rock salt, multiphase, and others). Among the models tested, XGBoost achieved the best performance with an F_1_-score of 86%, and the best-performing database variant combined primary (atomic and thermodynamic) features with statistical features. Finally, a neural-network classifier was trained using this most promising database variant in a more computationally intensive workflow focused on binary discrimination between perovskite and nonperovskite phases, yielding an F_1_-score close to 98% in an independent test set that was never seen during training, whereas the traditional Goldschmidt tolerance factor correctly classified only 67.3% of the same test samples. To open this black-box model, analyses based on the SHAP framework (SHapley Additive exPlanations) were carried out and links with the physics of HEOs were established, thereby providing the model with meaningful physical interpretation.

Overall, the combined use of machine learning and large language models has the potential to significantly advance the still underdeveloped field of HEO design.^29^ By enabling the extraction of latent knowledge from literature-derived HEO data,^30^ this framework supports not only the construction of accurate predictive models, but also the identification of physically meaningful design rules through model-interpretability techniques. Accurate phase prediction is particularly important in HEOs because many applications are phase-specific, with the relevant functional properties being strongly correlated with crystal structure.^3^ More broadly, the methodology developed here is also expected to be transferable to other classes of advanced materials that suffer from analogous data-scarcity limitations, thereby expanding the practical scope of machine learning in materials discovery.

## Methodology

The methodology adopted in this study is schematically illustrated in Figure 1. It was applied to discover new HEO compositions with a desired target phase, aided by machine learning and large language models. The process begins with the use of tools to extract data from the scientific literature, followed by the construction of a database that enables the training of machine learning classification models. Subsequently, the models are evaluated using typical performance metrics, and the SHAP library is utilized to provide insights into the latent knowledge abstracted from the data patterns by the models during training. Details regarding each step are provided below.

**Figure 1:**
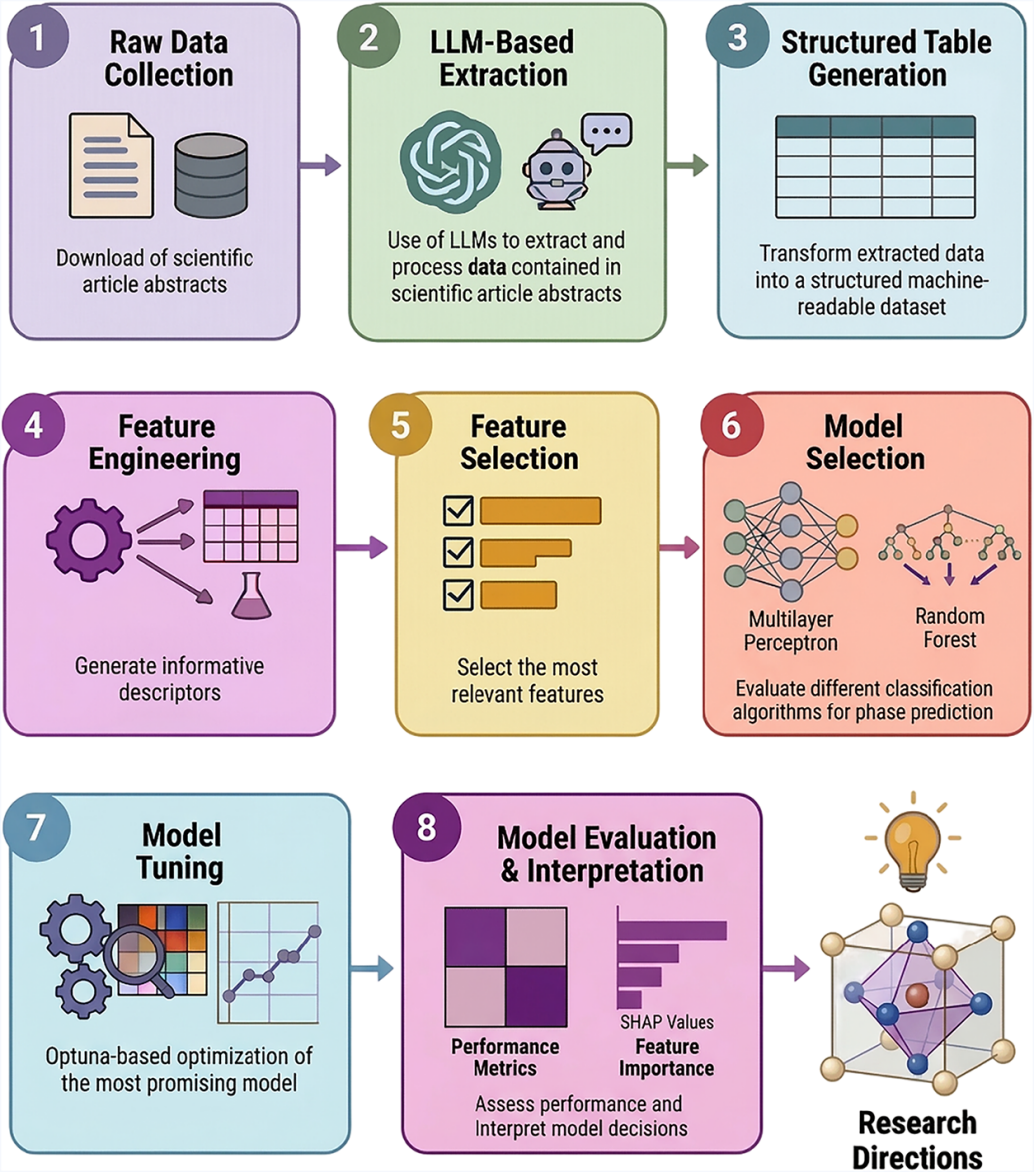
Overall workflow adopted in this study for phase-targeted high-entropy oxide design: (1) raw data collection, (2) LLM-based extraction, (3) structured table generation, (4) feature engineering, (5) feature selection, (6) model selection, (7) model tuning, and (8) model evaluation/interpretation.

### Data Extraction and Availability

Initially, 4432 abstracts of scientific articles were retrieved from *OpenAlex* on June 25, 2025, using the advanced search query “*high entropy” AND* “oxide*”. The processing of abstracts was carried out by using two different agents developed within the gpt-oss-120b framework. The first agent was tasked with associating and extracting the compositions, phases, and synthesis methods of HEOs, where the composition is a string written in standard chemical notation, the phase is a string representing the crystal system, and the synthesis method corresponds to the experimental technique used for material preparation. The second agent was responsible for receiving the extracted composition and phase and returning a vector containing the relative proportions of each cation in the composition. This is not a trivial task, as this calculation requires an understanding of both the number of sites and the number of substituting species at each site. Performing this second task without an agent for hundreds of data points would also be extremely cumbersome, as doing it manually would be highly time-consuming. In addition, doing it heuristically (by programming) would be prone to many errors, since it is not possible to completely interpret chemical formulas using regex, and in many cases, for HEOs, relying solely on the written chemical formula does not allow one to accurately determine the structure’s sites and their atomic occupancies.

A manual verification of 50 random samples from the data set was carried out to assess the accuracy of the two created agents. Furthermore, the final instructions, that is, after an iterative refinement, provided to the agents are available in the Supporting Information. It is worth mentioning that the prompts provided in Boxes S1 and S2 of the Supporting Information not only offer detailed instructions and unambiguous definitions but also require the agent to justify its answers, which enabled high accuracies even on nontrivial tasks.

The extracted data is available at the Materials Informatics Database (www.midb.cloud), which is created and maintained by some of the authors of this paper. We have implemented the digestion of natural language processing extraction data, from a json format we named as “.nlpe”; the link for all data and specifications can be found in the *Data Availability Statement* section.

### Feature Engineering

For the calculation of the primary features, we used three different data sources: (1) atomic properties provided by the NOMAD Lab (The Novel Materials Discovery Laboratory);^31^ (2) thermodynamic reaction data extracted from FactSage;^32^ and (3) key attributes associated with the formation of stable phases in high-entropy oxides, as described in the literature,^9,32–35^ with the necessary data sourced from the Mendeleev library.^36^ For the vectorization of these feature sets, we used a method similar to that proposed by Isayev et al.^37^ This method is based on the chemical composition of a compound, calculating the features from the individual properties of each element present while considering their relative proportions in the composition. Mathematical operations, such as maximum, minimum, standard deviation, mean, and sum, are then applied to transform the resulting vector into a single representative numerical value.

In the case of data obtained from FactSage, similar vectorization procedures were implemented, with calculations based on the formation equations of oxides under standard temperature and pressure conditions. For each equation presented in Table S1 (Supporting Information), the software calculated the corresponding values for entropy change, enthalpy, Gibbs free energy, and specific heat. In total, 246 primary features were constructed, and their detailed description can be found in Figures S1 and S2 (Supporting Information).

From the primary feature data set, two additional data sets were generated by incorporating features derived from a hot encoding of the synthesis method, together with statistical and interaction-based descriptors. The first enriched data set includes advanced statistical features such as covariance (features with suffix _cov), ratios (_min_to_max_ratio), and range (_range), combined with encoded synthesis labels.

The second engineered interaction data set builds on the enriched data set by introducing synthesis interaction features in addition to statistical descriptors. These features are generated by multiplying existing data features with synthesis group columns (syn_group_name). This operation assigns a weight to each synthesis method based on the magnitude of a given feature, ensuring that synthesis routes influence the model beyond conventional one-hot encoding.

All synthesis interaction features follow the naming convention [Feature Name]_x_synth_[Synthesis Method] and constitute the majority of the data set columns. Three main groups of synthesis interaction features were generated:

1. **Thermodynamic and energetic properties** that capture the energetic feasibility of phase formation under different synthesis conditions. Representative examples include:
  - Enthalpy × combustion synthesis
  - Gibbs free energy × hydrothermal synthesis
  - Melting point × solid-state synthesis
  - Boiling point × solvothermal synthesis
2. **Atomic and electronic properties** that describe bonding behavior and preferred crystal structures, which may vary with synthesis routes. Examples include:
  - Electronegativity × coprecipitation synthesis
  - Atomic radius × ball-milling synthesis
  - Valence electron concentration × hydrothermal synthesis
3. **Physical and mechanical properties** that reflect material transport, mixing efficiency, and energy requirements during synthesis. Examples include:
  - Solid density × mechanochemical synthesis
  - Thermal conductivity × combustion synthesis

Together, the data set containing only primary features and the enriched and engineered interaction data sets are referred to as 1_ds, 2_ds, and 3_ds, respectively, in the text below. These multiclass data sets classify materials into seven categories—spinel, perovskite, fluorite, pyrochlore, rock salt, multiphase, and others—with a pronounced imbalance: spinel dominates (33%), followed by perovskite (19.7%) and fluorite (18.3%), while the remaining classes each account for approximately 7.5%. Figure S3 provides a histogram that illustrates these class distributions (Supporting Information).

### Feature Selection

All three data sets were initially preprocessed through a standardized pipeline that included imputing missing values and encoding categorical features to ensure consistency across inputs. From each of these preprocessed data sets, three additional reduced-feature versions were generated using distinct feature selection strategies:

1. **Filtering approach** applied supervised selection of original and one-hot encoded features through a three-step process: (i) a low-variance filter with a threshold of 0.01, which removed nearly constant features unlikely to contribute to predictive modeling; (ii) correlation pruning, where features were standardized and a correlation matrix was computed to eliminate one feature from any pair exceeding a correlation threshold of 0.90, thereby reducing redundancy; and (iii) mutual-information-based supervised selection to identify the remaining features that contribute most strongly to class prediction. In this step, the target variable was label-encoded, and the mutual information between each feature and the class label was estimated using a nonparametric *k*-nearest-neighbors approach. Features were ranked according to their information gain, and the top-*K* features were retained. The value of *K* was defined as a fixed fraction of the postcorrelation feature set, subject to an upper bound.
2. **Dimensional reduction approach** employed unsupervised projection of all preprocessed features into principal components using PCA, generating up to 50 components, constrained by either the maximum possible or a user-defined limit.
3. **Combined approach** integrated both strategies by first executing the filtering pipeline and then applying PCA to the reduced set, thereby balancing supervised feature relevance with unsupervised dimensionality reduction.

In the filtered data sets, the number of retained features varied by source: 1_ds contained 13 features, 2_ds contained 21 features, and 3_ds contained 44 features. The complete lists of selected features are provided in Tables S2–S4 (Supporting Information). For the dimensional reduction data sets, 50 principal components were consistently created, while the combined data sets preserved the same feature counts as their filtered counterparts but expressed them in PCA-transformed space.

This structured preprocessing and feature engineering framework ensured that each data set variant captured different balances of interpretability, redundancy reduction, and dimensional compactness, enabling robust experimentation across supervised and unsupervised modeling pipelines. The resulting data sets were systematically labeled X_ds_Filter_features, X_ds_DimenReduct_features, and X_ds_Combin_features.

### Training Machine-Learning Models

We began by training general-purpose exploratory models to distinguish among the different phase classes of HEOs, using multiple variations of the data set as input. For this purpose, both the basic data sets and the newly created reduced-feature variants were used to train a diverse set of machine-learning models, including XGBoost, LightGBM, CatBoost, Random Forest, SVC with an RBF kernel, and Logistic Regression.

Building on insights from this initial stage, we subsequently trained a Multilayer Perceptron (MLP)^38^ tailored for binary classification, specifically distinguishing perovskites from nonperovskites, thereby focusing on the predictive structure of this key material class.

#### Multiclass Classification

For the multiclass classification task, a total of 344 samples was used. As shown in Table 3, the multiclass data set variants differ in the number and type of features, but not in the total number of samples. Given the number of data set variants and models to be evaluated, a 1 × 5-Fold Tuning strategy was adopted to balance computational efficiency with robust evaluation. The data were initially partitioned into training, validation, and test sets using a 3:1:1 ratio, resulting in 206 training samples, 69 validation samples, and 69 test samples. The training set was then stratified into 5-fold for hyperparameter optimization via randomized search.

Model performance was primarily assessed using the F1-macro score, which captures precision-recall trade-offs across imbalanced classes. To resolve ties and provide complementary information, balanced accuracy (maximum), log loss (minimum), and Brier score (minimum) were employed as secondary metrics.

The tuned pipelines were first obtained through randomized search with 5-fold cross-validation on the training set. These tuned pipelines were then fitted to the full training set and evaluated once on the validation set. Finally, the optimized pipelines were refitted on the combined training and validation sets and subjected to a single evaluation on the test set, ensuring an unbiased and rigorous assessment of the generalization performance.

#### Binary Classification

For the binary classification task, the synthesis method variable was removed because it showed low relevance in the exploratory stage; this also allowed an increase in the number of usable samples. Under this formulation, the binary classification task consisted of a total of 711 samples. Among the data set variants evaluated, the *Enriched Binary Data set* (Table 3) was selected for this task because it showed the most promising performance, as discussed in the Results and Discussion section. The data were split into training and test sets using an 80/20 ratio, resulting in 568 training samples and 143 test samples. Hyperparameter tuning was performed within the training set using 5-fold cross-validation.

A neural network model was adopted both because of the larger sample availability in the binary setting and because of its demonstrated capacity to achieve strong predictive performance across a wide range of applications.^39^ The model was initially trained using all features from the enriched data set. Hyperparameter optimization was then carried out with the Optuna framework, which employs Bayesian optimization techniques.^40^ In total, approximately 20 hyperparameters were tuned (further details are provided in Table S5 of the Supporting Information). Subsequently, the SHAP framework was applied to rank the features according to their importance, and a series of models was trained using feature subsets ranging from 6 to 90 features, in increments of 7, in a grid-search procedure. After the optimal number of features were identified, the model underwent a second stage of hyperparameter optimization, again using Optuna, resulting in the final fully optimized architecture. The perovskite phase was defined as the positive class because of its high potential for application in SOFC electrode materials.^8,41–43^

#### Evaluation Metrics

The metrics used for model evaluation are presented in eqs 2–7 and correspond, respectively, to accuracy, F1-macro, ROC AUC, PR AUC, precision, and recall.

2 $$a\mathrm{ccuracy}=\frac{\mathrm{TP}+\mathrm{TN}}{\mathrm{TP}+\mathrm{TN}+\mathrm{FP}+\mathrm{FN}}$$

3 $$p\mathrm{recision}=\frac{\mathrm{TP}}{\mathrm{TP}+\mathrm{FP}}$$

4 $$r\mathrm{ecall}=\frac{\mathrm{TP}}{\mathrm{TP}+\mathrm{FN}}$$

5 $$F1-\mathrm{macro}=\frac{1}{N}\sum_{i=1}^{N}\frac{2\cdot {p\mathrm{recision}}_{i}\cdot {r\mathrm{ecall}}_{i}}{{p\mathrm{recision}}_{i}+{\mathrm{recall}}_{i}}$$

6 $$\mathrm{ROCAUC}=\int_{0}^{1}TPR\left(FPR\right)d\left(FPR\right)$$

7 $$\mathrm{PRAUC}=\int_{0}^{1}P\left(R\right)\mathrm{dR}$$

where TP, TN, FP, and FN denote true positives, true negatives, false positives, and false negatives, respectively. The AUC-based metrics represent the area under the ROC and precision-recall curves, respectively.

Considering the pronounced class imbalance in the multiclass classification setting, we employed additional metrics to analyze the models during the exploratory phase, namely, balanced accuracy, Brier score, and log loss, calculated according to eqs 8–10.

8 $$b\mathrm{alanced}\;a\mathrm{ccuracy}=\frac{1}{K}\sum_{k=1}^{K}{r\mathrm{ecall}}_{k}$$

9 $$b\mathrm{rier}\;s\mathrm{core}=\frac{1}{N}\sum_{i=1}^{N}\sum_{k=1}^{K}{\left(p_{i,k}-o_{i,k}\right)}^{2}$$

10 $$l\mathrm{og}\;l\mathrm{oss}=-\frac{1}{N}\sum_{i=1}^{N}\log\left(p_{i,y_{i}}\right)$$

where *p*_*i*,*k*_ is the predicted probability for class *k*; *o*_*i*,*k*_ is the one-hot encoded true label, and *p*_*i*,*y*_*i*__ is the predicted probability assigned to the true class *y*_*i*_. To provide a robust statistical evaluation of the metrics measured in the test set, confidence intervals were computed by using the bootstrap method. This approach involved generating new data sets from the test set through random sampling with replacement. For each metric, a total of 2000 bootstrap samples were created and the confidence interval was determined using the percentile method, as implemented in the Python library NumPy.^44^ A 95% confidence level (α = 0.05) was applied. The confidence interval is described in eq 11, where the array contains the metric values computed for each bootstrap sample.

11 $$\mathrm{CI}=\left[{p\mathrm{ercentile}}_{1-\alpha /2}\left(\mathrm{array}\right),\;{p\mathrm{ercentile}}_{1+\alpha /2}\left(\mathrm{array}\right)\right]$$

### SHAP: Feature Selection and Model Explainability

The Python library SHAP^45^ (SHapley Additive exPlanations) is grounded in game theory and can be utilized for both feature selection and gaining insights into how models make decisions (black-box model explainability).

Since the SHAP framework interprets features as players in a cooperative game, it has greater potential to identify an optimal set of features for enhancing model performance compared to other feature selection methods.^46^ To fairly quantify the relative contribution of each feature within a group of features, SHAP evaluates the model’s outputs across all possible feature coalitions within defined limits, as expressed in eq 12.

12 $$g\left(z^{\prime }\right)=\varphi_{0}+\sum_{j=0}^{M}\varphi_{j}z_{j}$$

where *g* represents the explanation model, *z*′ is the coalition vector, *M* is the maximum size of a coalition, and ϕ_*j*_ is the impact value of the feature in the model. ϕ_*j*_ is a real number, and *z*′ ∈ {0, 1}^*M*^. If ϕ_*j*_ has a high positive value, it indicates that feature *j* significantly contributes to supporting a given class prediction by the model. In contrast, if ϕ_*j*_ has a high negative value, it implies that feature *j* strongly contributes to rejecting the class prediction. Finally, if ϕ_*j*_ is close to 0, the feature *j* has little to no contribution to the model’s decision-making process. For the binary classification neural network, SHAP values were employed both for feature selection and for the final interpretability of the model. The visualizations generated by the library provide highly informative insights into the motivations and tendencies underlying the model’s decisions. This enables a rational understanding of the latent and abstract knowledge that the models captured from the HEOs data sets during training.

## Results and Discussion

All performance metrics and their confidence intervals for the best models obtained for both the multiclass phase classification and binary classification tasks are reported in Table 1. As expected, the metrics for the multiclass task are lower, since it is a more challenging problem; nevertheless, in both settings, the scores are numerically close to 1, indicating good generalization ability and, therefore, effective abstraction of latent knowledge from the patterns present in the data sets.

**Table 1:** Performance of the Best Models for Multiclass (XGB) and Binary (MLP) Classification

**model**	**metric**	**value**	**95% confidence interval**
XGB	accuracy	0.899	[0.826, 0.957]
	F1-macro	0.858	[0.747, 0.942]
	ROC AUC	0.950	[0.903, 0.989]
	PR AUC	0.886	[0.797, 0.968]
	precision	0.874	[0.793, 0.959]
	recall	0.862	[0.760, 0.953]
MLP	accuracy	0.979	[0.951, 1.000]
	F1-macro	0.977	[0.946, 1.000]
	ROC AUC	0.999	[0.995, 1.000]
	PR AUC	0.997	[0.991, 1.000]
	precision	0.940	[0.870, 1.000]
	recall	1.000	[1.000, 1.000]

As outlined in the Methodology section, we adopted an exploratory strategy in the multiclass classification stage that enabled training multiple models across several data set variants. This approach not only yielded strong multiclass classifiers—most of them based on tree-based algorithms, which often perform competitively with neural networks, particularly in smaller data sets^47^—but also allowed us to identify the most promising data set variant.

In the second stage, that enriched data set variant was selected and a more complex model (MLP) was trained using a more computationally demanding and time-consuming procedure (also detailed in the Methodology), since artificial neural networks tend to achieve superior performance compared to other models, especially on larger data sets.^39^ For the binary classification task in this second stage, the perovskite phase was defined as the positive class to be distinguished from all other phases, as perovskites are the phase of primary interest due to their potential to mitigate the trade-off between chemical stability and catalytic activity in the SOFC electrode.^8^

### Multiclass Classification

Given the imbalance between classes, model performance across all data sets was evaluated primarily using the F1-macro score (Figure 2a), which summarizes the precision-recall trade-off for each model. Accuracy and balanced accuracy (Figure 2b) were additionally considered to assess the extent to which models exploit class imbalance. Further evaluation was conducted using Brier scores and log loss (Figure S4), both of which measure probability- and error-based performance.

**Figure 2:**
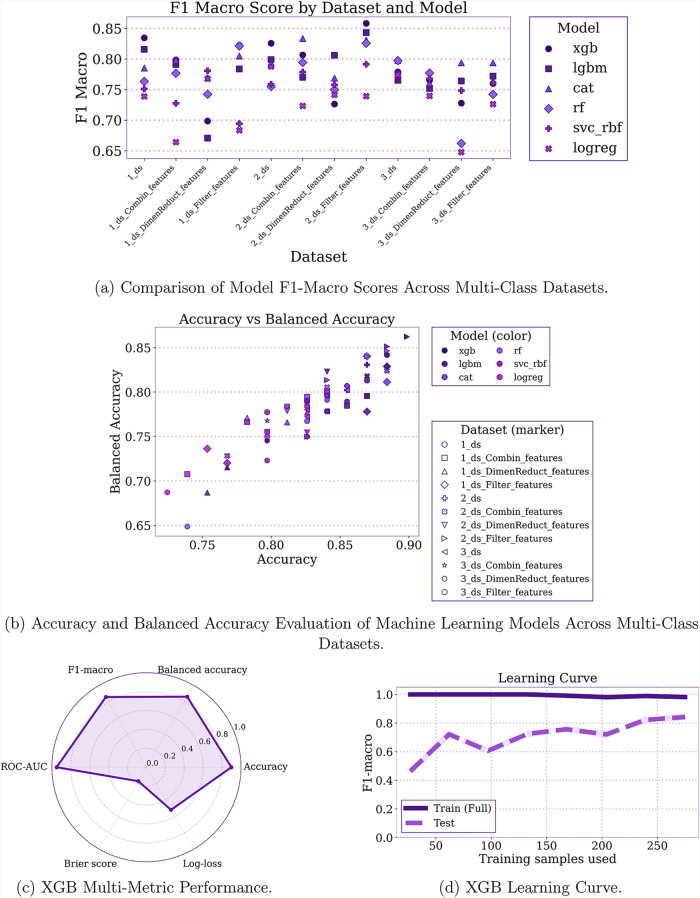
Multiclass classification overview across data sets and models. Panels summarize: (a) F1-macro performance, (b) accuracy versus balanced accuracy, (c) multimetric performance for the best-performing XGBoost configuration, and (d) learning curve also for XGB.

Across most data sets, gradient-boosted decision tree models (XGBoost, CatBoost, and LightGBM) achieved the highest F1-macro scores and balanced accuracies, demonstrating strong capability in capturing minority-class patterns. These models also produced the lowest Brier scores and logarithmic loss values, indicating reliable probability estimates. In contrast, Logistic Regression consistently ranked the lowest across all metrics. Its poor F1-macro and probability performance, combined with uniformly low accuracy and balanced accuracy, suggest that the model performs poorly across all classes rather than disproportionately favoring the majority class.

At the data set level, the data sets combining synthesis methods with specific properties (group 3_ds) produced relatively narrow distributions of F1-macro scores across models, indicating moderate learnability even for complex architectures. This group of data sets also consistently yielded the lowest overall F1-macro values (<0.80). In contrast, data sets constructed with PCA-derived features showed medium to low performance. This is likely because the principal components maximize the linear variance in the data and compress the feature space into linear combinations. Such transformations fail to preserve nonlinear dependencies or higher-order interactions among physical properties. Consequently, physically meaningful feature boundaries may be obscured, and reduce the ability of models—particularly tree-based algorithms that depend on axis-aligned, nonlinear splits—to identify discriminative patterns. In contrast, data sets enriched with basic and advanced statistical descriptors, whether raw or filtered (1_ds, 1_ds_Filter_features, 2_ds, 2_ds_Filter_features), achieved stronger results, reflecting their greater capacity to preserve informative signals.

In all data sets and models, the 2_ds_Filter_features data set (containing advanced statistical descriptors with filtered features) combined with the XGBoost model achieved superior performance (Figure 2c). It obtained the highest F1-macro score, exhibited a minimal discrepancy between accuracy and balanced accuracy, and produced the lowest Brier score and log-loss value.

The filtered feature data set removed nearly constant variables that contributed little to variance and eliminated highly correlated features that introduced redundancy. The inclusion of advanced statistical descriptors enriched the data set with alternative but complementary representations of the same underlying physical properties. This expansion enabled mutual information to assess a broader range of relationships with the target class, helping to identify which basic features carried the true signal. Although the retained subset after filtering was largely composed of basic statistical descriptors (Table S3), the presence of advanced features increased the opportunity to preserve highly informative variables.

Through supervised selection, the final subset emphasized variables that captured nonlinear and higher-order dependencies with the target classes. This systematic reduction of irrelevant and redundant features reduced the noise propagated in gradient-based updates, allowing the XGB model to construct more stable and meaningful decision splits. As a result, the model achieved higher F1-macro scores, reflecting balanced improvements across all classes while narrowing the gap between accuracy and balanced accuracy. Probability calibration also improved, as evidenced by reduced Brier scores and log-loss values, indicating that the predicted probabilities aligned more closely with the true class distributions.

The learning curves for F1 macro (Figure 2d) show that XGB fits the training data nearly perfectly but begins to generalize reasonably well once approximately 100 samples are available. The persistent train-test gap of roughly 15% suggests that, despite filtering, the data set remains complex enough that the model cannot fully capture all decision boundaries with limited data. This generalization behavior is further supported by the confusion matrix (Figure S8 in the Supporting Information), which demonstrates that the model learned discriminative patterns for classes with more distinctive properties (fluorite, perovskite, rock salt, and spinel), while minor misclassifications occurred in underrepresented classes (multiphase, pyrochlore, and others) where chemical and structural distinctions are less pronounced. Overall, these results confirm that most classes in the data set enriched with advanced statistical features are well separated following feature filtering. Despite some degree of overfitting to the training data, the XGBoost model established well-calibrated decision boundaries, yielding accurate class predictions with only sparse and chemically plausible misclassifications. Consistent with previous studies on various chemical and biological prediction tasks,^48–50^ XGBoost is known to fit training data extremely tightly. This tendency toward overfitting is evident when comparing training and test-set performance; however, the model’s predictive accuracy remains superior to, or at least competitive with, the best available alternatives.

### Binary Classification

Figure 3a,b show the ROC and PR curves for the multilayer perceptron, with ROC AUC = 0.999 and PR AUC = 0.997. These values demonstrate an excellent ability to distinguish test samples (kept entirely unseen during training) as perovskite or nonperovskite using only computationally inexpensive features, indicating strong generalization to new data. This conclusion is further supported by the confusion matrix in Figure 3c, which shows only 3 misclassifications out of 143 test samples. Together, these results underscore the promise of machine-learning-based techniques for exploring multicomponent material spaces, which cannot be probed by traditional approaches alone, as the number of possible compositions can reach an order of 10^46^.^4^

**Figure 3:**
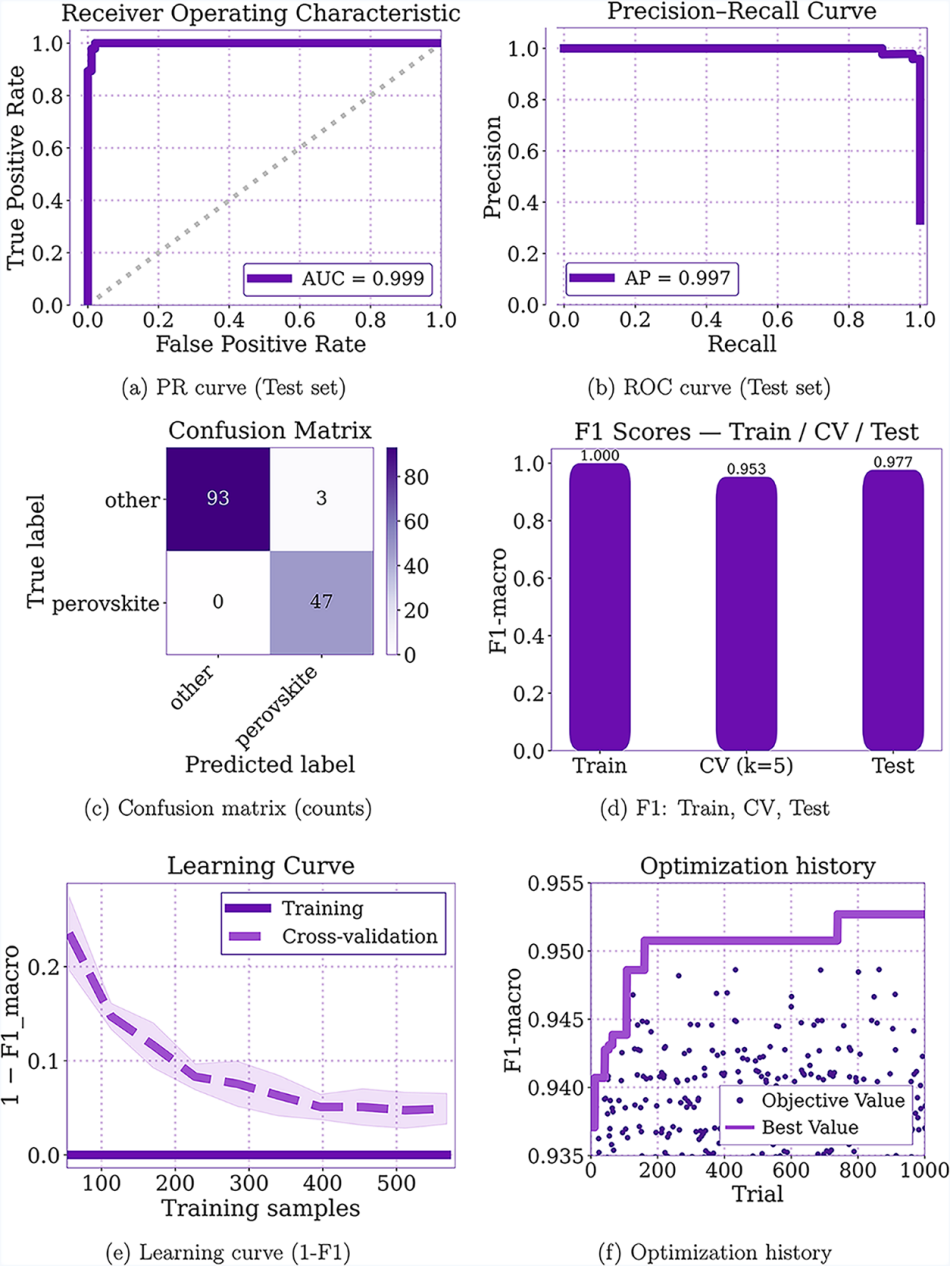
(a–e) Results on the test set: ROC/PR curves, confusion matrix, F1 (Train/*C–V*/Test), and learning curve. (f) Optimization history.

Due to the class proportion of approximately one-third perovskite and two-thirds other phases, as shown in Table S6 of the Supporting Information, the main performance metric considered was the macro-averaged F_1_ score (F_1_-macro). Figure 3d shows that the obtained values are numerically very close to 1 for all three splits (training, cross-validation, and test), indicating no evident overfitting. To examine this more closely, a learning curve was constructed as shown in Figure 3e, showing how increasing the number of training samples moves the model from a potential overfit regime (fewer than 400 training samples) to a regime without overfitting, where the cross-validation and training curves are closely aligned.

Figure 3f shows the optimization history generated by the Optuna framework. Although 12,000 trials (hyperparameter combinations) were performed, no further improvement in the cross-validated *F*_1_-macro score was observed after approximately the 739th trial, indicating that the optimization process was carried out with more than a sufficient number of trials. The final selected hyperparameters are reported in Table S5 of the Supporting Information.

The fANOVA analysis revealed that the solver was the most important hyperparameter (see Figure S6 in the Supporting Information), which is a consistent result, since several other hyperparameters are only accessible depending on the chosen solver, making the solver hierarchically dominant. We have also included in the Supporting Information (Figure S5) a parallel-coordinates plot showing the best solutions and their corresponding hyperparameters. In particular, the best-performing configurations were obtained using the Adam solver with a single hidden layer, and the regularization parameter α exhibited substantial flexibility, admitting a wide range of values among the top solutions.

To conclude the performance analysis in a robust manner, Figure 4f visually summarizes all performance metrics and their respective confidence intervals in the form of a radar chart. The innermost circle of the radar is set to 0.86, showing that even the lower bounds of the confidence intervals are numerically close to 1, which further reinforces the model’s ability to generalize and produce reliable inferences. Therefore, the screening of compositions across the virtually infinite design space of high-entropy oxides can be carried out efficiently using appropriately trained machine-learning models.

**Figure 4:**
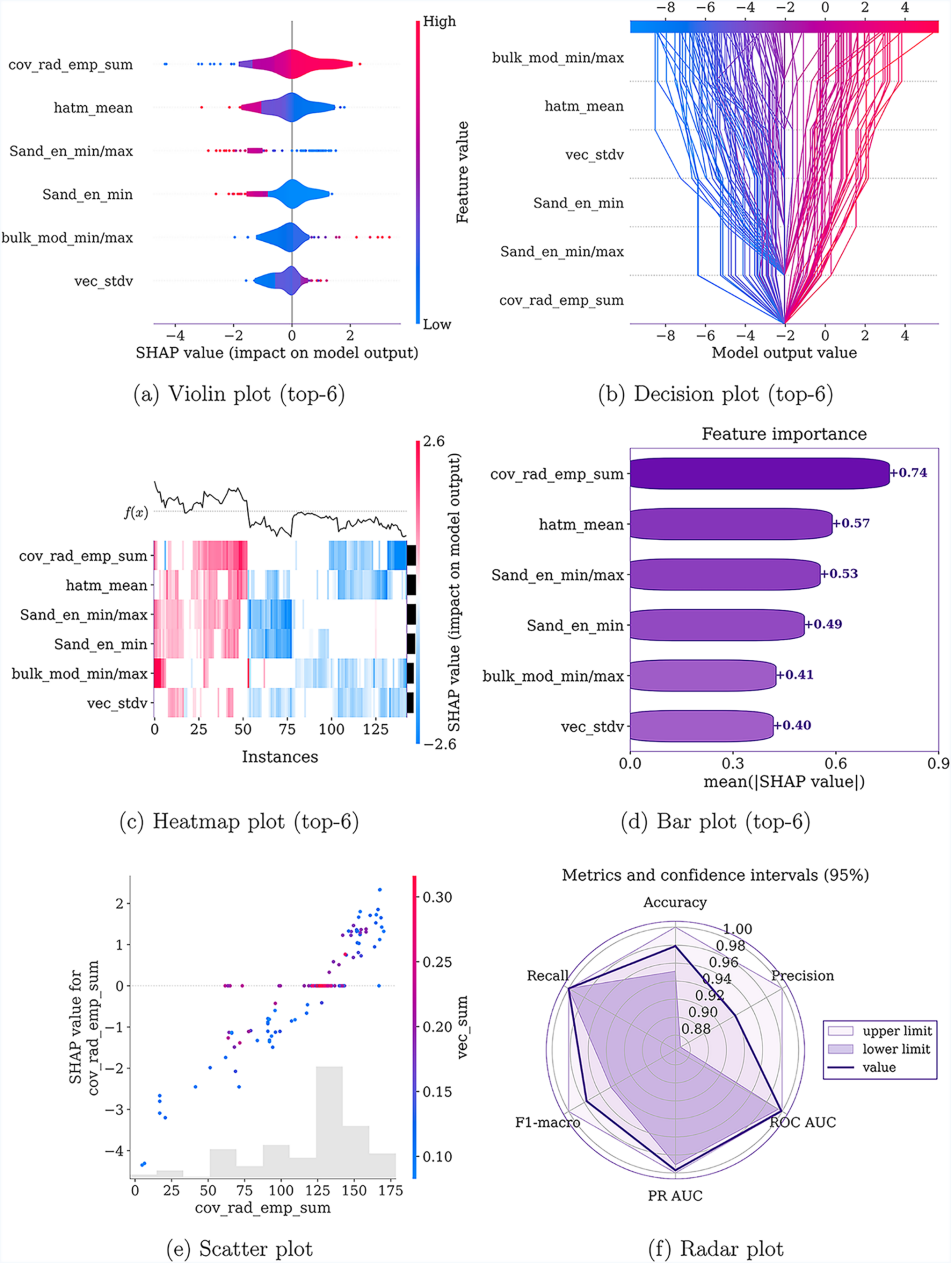
Panels (a–e) correspond to SHAP-based explainability plots, whereas panel (f) shows a radar plot of the performance metrics with their confidence intervals.

Beyond predictive modeling, machine-learning methods, when combined with explainability frameworks, enable the extraction of physical insights into the studied phenomenon. In this context, Figure 4a–e presents the MLP interpretability plots for the six most important features, and the definitions of the physical properties underlying these descriptors are provided in Table 2. The complete list of all 55 features used in the model is provided in Table S7 of the Supporting Information. In Figure 4d, the global mean contribution of the most important features is shown, which together account for approximately 40% of the total decision weight, and the remaining 60% is distributed across the other 50 features. These six features are therefore learned as key descriptors governing stabilization of the perovskite phase in high-entropy oxides and merit detailed analysis to extract physical insights into the underlying phenomenon, given that latent knowledge in the data patterns has been abstracted by the model during training.

**Table 2:** Definitions of the Features Selected by SHAP for the MLP (Top-6)

**feature**	**vectorization operation**	**definition**
cov_rad_emp_sum	sum over all values	covalent radius
hatm_mean	mean value	enthalpy of atomization
sand_en_min/max	min/max quotient	sanderson electronegativity
sand_en_min	minimum value	sanderson electronegativity
bulk_mod_min/max	min/max quotient	bulk modulus
vec_stdv	standard deviation	valence electron concentration

To more clearly elucidate how these features control the stabilization of the perovskite phase in high-entropy oxides, Figure 4a shows how their values drive the model’s decision to assign or reject the perovskite class. Notably, the sum of the cation radii is identified as the most relevant factor, which is physically consistent with expectations for the perovskite structure, which is known to be more flexible and able to accommodate larger cations than other crystal structures.^51,52^

Descriptors for predicting perovskite formation in high-entropy oxides frequently rely on elemental electronegativity.^53^ As shown in Figure 4a–d, in our model the minimum and maximum Sanderson electronegativity values, as well as their ratio, exert a particularly strong influence on perovskite phase formation. This is a noteworthy result, given that the Sanderson electronegativity scale has not previously been employed to predict the structural stability in high-entropy perovskites. Although different electronegativity scales may exhibit similar qualitative trends, their predictive capabilities can differ substantially.^54^ It is well established that because there is a strong correlation between cation electronegativity and compound polarity, the polarization state arising from electronegativity differences exerts a substantial influence on the crystal structure of the material. Consequently, the crystal structure of HEOs is, at least from a theoretical standpoint, expected to be closely linked to the electronegativity of the cations, which is consistent with the SHAP-based feature-importance results.^16^

Discussions of phase stability in high-entropy oxides frequently invoke the Gibbs free energy of mixing, Δ*G*_mix_ = Δ*H*_mix_ – *T*Δ*S*_mix_.^55^ However, the explicit evaluation of these thermodynamic quantities is computationally demanding and is not straightforward. As shown in Figure 4a–d, our model instead identifies an enthalpic descriptor that is inexpensive to compute yet reflects both the bond energy and the effective number of bonds associated with the cations, and is therefore indirectly related to the mixing enthalpy. In particular, a low composition-averaged cation atomization enthalpy, hatm_mean, is predicted in Figure 4a to favor the formation of the perovskite phase.

Consistent with Figure 4a, the model also indicates that a low standard deviation of the valence electron concentration among the cations disfavors the formation of the perovskite phase. The valence electron concentration (VEC) is a widely used descriptor to rationalize phase stability in high-entropy materials,^9^ further strengthening the credibility of the trends captured by our model. From a physical point of view, valence electrons largely determine the topology and occupancy of chemical bonds in the solid and are therefore directly related to the stability of competing crystal structures; as a consequence, each phase tends to stabilize within a characteristic VEC window.^56^ The model further suggests that large differences between the bulk moduli of the constituent cations favor the formation of the perovskite phase, which is likely related to the structural flexibility of the perovskite lattice.^51,52^ Although the bulk modulus is directly correlated with the valence electron concentration (VEC),^57^ to the best of our knowledge, it has not previously been used as an explicit descriptor to predict the formation of high-entropy oxides.

Figure 4b shows the individual impact of each feature on the classification of the test-set samples. The strong influence of both the sum of the cation radii and the electronegativity ratio between the minimum and maximum values on the test classifications (which achieved 97.9% accuracy) is evident. For example, in samples where the feature covalent_rad_emp_sum drives the decision toward the perovskite class, the remaining features rarely succeed in pushing the model to classify the sample as nonperovskite. This behavior further reinforces the reliability of these descriptors in predicting previously unseen data.

To assess the consistency of the model’s predictions on the test set, we show the SHAP heatmap in Figure 4c. The top row of the plot shows the model output, corresponding to the binary classification, which can be positive (perovskite) or negative (nonperovskite). A clear separation between the perovskite and nonperovskite regions is directly observed on the *Intances* axis. In the positive region, each descriptor almost invariably contributes to the perovskite class or remains with a low impact. The same behavior is seen in the nonperovskite region, where the descriptors across the predictions nearly unanimously support the nonperovskite classification or remain neutral. This pattern confirms that the structures learned by the model are consistent and reinforces the robustness of the proposed approach.

To further investigate the identified main descriptor, cov_rad_emp_sum, Figure 4e shows a SHAP interaction scatter plot capturing how this primary descriptor interferes with the descriptor exhibiting the strongest nonlinear interaction with it, that is, vec_sum. The plot reveals a clear trend: as the valence electron concentration increases to high values, cov_rad_emp_sum ceases to play a decisive role in determining whether a sample is classified as perovskite or nonperovskite. This behavior suggests that geometric aspects (i.e., cation radii) are no longer the dominant factor governing phase formation when the valence electron concentration is high. In this regime, electronic effects become more relevant for stabilizing the perovskite phase in high-entropy oxides.

To validate this result—namely that the geometric factor ceases to be decisive for certain cation combinations in stabilizing high-entropy oxide perovskites—we employed the well-established Goldschmidt tolerance factor to classify the compositions in our test set and assess how this geometric descriptor performs in perovskite classification, in a manner analogous to the analysis of conventional (low-entropy) perovskites by Bartel et al.^58^ We find that for high-entropy materials, the Goldschmidt tolerance factor attains an accuracy of 67.3% in distinguishing perovskite from nonperovskite phases, which is slightly lower than the 74% reported by Bartel et al.^58^ for conventional perovskites. By comparison, our machine-learning model achieves an accuracy of 97.8% on the same test set. These results indicate that traditional descriptors have reduced predictive power for high-entropy materials relative to conventional systems,^9^ and they simultaneously highlight the effectiveness of machine-learning models in capturing complex phase-formation phenomena, making them a preferable approach over criteria based solely on descriptors.

### Databases and LLM-Based Agent Evaluation

Table 3 summarizes the variants of the data set considered in this work, highlighting the type of features computed in each group. The base data set for the multiclass classification task contains fewer samples because we required the synthesis method to be explicitly reported in the abstract; as a result, the number of samples is smaller than in the data set used for binary classification. The decision to exclude the synthesis method as a feature from the binary classification models was motivated not only by the possibility of increasing the data volume but also by the observation that the synthesis method contributed little to the models’ decision-making, as demonstrated in Figure S7 in the Supporting Information. This limited relevance has a straightforward explanation: in the high-entropy oxide literature, cases in which the same composition is reported with different phases arising from distinct synthesis routes are exceedingly rare, and thus the statistical significance of the synthesis method is inherently low. Hence, as there are not sufficient data available, the model is unable to capture the importance of the synthesis method; thus, more systematic studies in this field are needed to improve the understanding of the synthesis route in the final HEO phase.

**Table 3:** Main Features of the Created Databases

**data source**	**number of samples**	**number of features**	**main characteristic**
base multiclass data set	344	246	primary features
base binary data set	711	246	primary features
enriched binary data set	711	470	primary + advanced statistical features
enriched multiclass data set	344	513	primary + advanced statistical + synthesis features
interactions multiclass data set	344	2991	primary + advanced statistical + interaction + synthesis features

To assess the reliability of the constructed data sets, we manually audited 50 randomly selected samples. For this audit, we adopted a conservative two-outcome criterion: exactly correct when the entire composition was written as a chemical formula in a correct manner with explicit stoichiometric coefficients and was correctly associated with its phase and synthesis method and incorrect when any error, incompleteness, or ambiguity was present. In this evaluation, the accuracies obtained for the extraction agent and for the stoichiometric-fraction computation agent, both based on the gpt-oss-120B model, are reported in Table 4. The prompts and parameter configurations employed for these agents are detailed in the Supporting Information. For each prompt, in addition to specifying all relevant contextual information and clear task instructions, we also provide illustrative input–output examples accompanied by an interpretation note explaining how the example input is mapped to the corresponding example output. The stoichiometric-fraction agent completed its task straightforwardly under the given instructions, achieving 100% accuracy. The extraction and data-association agent reached 94% accuracy; however, after applying regular expressions to remove invalid compositions (e.g., strings containing characters that do not correspond to valid chemical elements), the accuracy of the effective data set increases to 96%. These results demonstrate that large-language-model-based agents can reliably construct databases of advanced materials, in line with the findings of Polak et al.^59^ They can do so even when complex operations are required, as in the work of Zimmermann et al.,^18^ such as computing relative cation proportions—a nontrivial task that demands understanding how atoms are distributed over the crystallographic sites of the structure.

**Table 4:** Performance of Each Agent Manually Computed Using 50 Random Samples

**task**	**accuracy**
extraction and association of composition, phase, and synthesis method	0.94
calculation of the relative proportions of each cation	1

## Conclusion

We have demonstrated an end-to-end, data-centric strategy for accelerating high-entropy oxide phase design by coupling large-language-model-based literature mining with machine-learning classifiers. Using gpt-oss-120B agents augmented by regular expressions, we programmatically extracted compositions, phases, and synthesis labels from *Web of Science* abstracts and reliably computed cation stoichiometric fractions. Manual auditing confirmed that this approach can generate complex and internally consistent HEO data sets with high fidelity, reaching an effective accuracy of 96% for extraction/association and 100% for stoichiometric-fraction computation. The resulting primary databases enabled systematic feature engineering and model training across multiple data set variants, providing a practical blueprint for overcoming the persistent data-scarcity bottleneck that limits machine-learning applications in HEOs research.

For the exploratory seven-class phase prediction, gradient-boosted tree models showed the most robust performance across imbalanced classes. In particular, XGBoost trained on the statistically enriched and filtered representation (2_ds_Filter_features) achieved the best overall generalization (F_1_-macro = 85.8%). Building on this outcome, we trained a dedicated MLP for binary perovskite versus nonperovskite discrimination using the best-performing enriched feature strategy. The final model achieved 97.9% test accuracy, substantially outperforming the traditional Goldschmidt tolerance factor baseline (67.3% accuracy) on the same test set. These results show that machine-learning models trained on inexpensive composition-derived descriptors can capture complex high-entropy phase-formation trends with a markedly higher predictive power than conventional rule-based criteria.

The combination of machine learning and SHAP interpretability also provided physically meaningful insights. The model highlighted a compact set of key descriptors, most notably the summed covalent radii, Sanderson electronegativity extrema, mean atomization enthalpy, bulk modulus contrast, and VEC dispersion, suggesting that high-entropy perovskite stabilization is governed by an interplay of geometric and electronic effects.

Future work should integrate active-learning loops that iteratively propose and experimentally validate new compositions. Extending this framework to other families of advanced materials that also suffer from data scarcity in typical material databases is also a natural next step. Overall, the methodology presented here establishes a scalable and reproducible pathway for large language model-enabled data set construction and machine-learning-guided discovery of single-phase HEOs, offering a practical route to navigate the vast compositional design space and to accelerate the rational development of phase-targeted high-entropy materials.

## Supplementary Material



## Data Availability

To ensure that this study can be reproduced and improved, Python scripts and the models trained in this article are accessible under the open-source MIT License on GitHub: https://github.com/Arthurns16/LLM-empowered-machine-learning-assisted-prediction-of-single-phase-HEOs. The extracted data is available at the Materials Informatics Database and can be accessed at https://midb.cloud/shared_collection?key=0941b0d281b866c7a6de887798aab097. In order to facilitate the sharing of data and its collection in databases, extractions are stored in text files with the extension *.nlpe. The extraction data is encoded in *.nlpe files according to the json format and is validated with the json-schema shown in the Supporting Information (Figure S9). The json-schema is also available under the GPLv3 license at https://github.com/MaterialsInformaticsDB/MIDb-Specs/blob/main/nlpe_schema.json.

## Notes

Tables and figures detailing the gpt-oss-120B-based agents developed in this work, the constructed data sets, and the machine-learning models trained in this study (PDF)
