# Supplementary material for: Rational Design of Single-Phase High-Entropy Oxides via Large Language Model Data Mining and Explainable Machine Learning
Source: J Chem Inf Model. 2026 Apr 25;66(9):5234–48. doi: 10.1021/acs.jcim.6c00752 (PMC13169342; doi:10.1021/acs.jcim.6c00752)
Supplement: Supplementary file 1 [file ci6c00752_si_001.pdf]

Supporting Information: Rational Design of Single-Phase High Entropy Oxides via Large Language Model Data Mining and Explainable Machine Learning

Arthur da Silva Sousa Santos,† Elena Stojanovska,‡ Antonio Augusto Alves Junior,¶ Amauri Jardim de Paula,§ Daniel Zanetti de Florio,† and James Moraes de Almeida*,¶,‖

†*Center for Engineering, Modeling and Applied Social Sciences, Federal University of ABC (UFABC), Av. dos Estados, 5001, Bangú, Santo André, São Paulo 09210-580, Brazil*

‡*Independent Researcher, Trento, TN 38122, Italy*

¶*Ilum School of Science, Brazilian Center for Research in Energy and Materials (CNPEM), Rua Lauro Vannucci, 1020, Fazenda Santa Cândida, Campinas, São Paulo 13083-970, Brazil*

§*Aeronautics Institute of Technology (ITA), Praça Marechal Eduardo Gomes, 50, Vila das Acacias, São José dos Campos, São Paulo 12228-900, Brazil*

‖*Center of Natural and Human Sciences, Federal University of ABC (UFABC), Av. dos Estados, 5001, Bangú, Santo André, São Paulo 09210-580, Brazil*

E-mail: james.almeida@ilum.cnpem.br

Table S1: Equations used in calculations with FactSage.

Cation	Equation	Cation	Equation
Er	$4\ Er + 3\ O_2 \rightarrow 2\ Er_2O_3$	Be	$2\ Be + O_2 \rightarrow 2\ BeO$
Sm	$4\ Sm + 3\ O_2 \rightarrow 2\ Sm_2O_3$	Sc	$4\ Sc + 3\ O_2 \rightarrow 2\ Sc_2O_3$
Eu	$4\ Eu + 3\ O_2 \rightarrow 2\ Eu_2O_3$	Pb	$Pb + O_2 \rightarrow PbO_2$
Tm	$4\ Tm + 3\ O_2 \rightarrow 2\ Tm_2O_3$	Ba	$2\ Ba + O_2 \rightarrow 2\ BaO$
Li	$4\ Li + O_2 \rightarrow 2\ Li_2O$	Ga	$4\ Ga + 3\ O_2 \rightarrow 2\ Ga_2O_3$
Dy	$4\ Dy + 3\ O_2 \rightarrow 2\ Dy_2O_3$	Co	$3\ Co + 2\ O_2 \rightarrow Co_3O_4$
Mn	$4\ Mn + 3\ O_2 \rightarrow 2\ Mn_2O_3$	Ta	$4\ Ta + 5\ O_2 \rightarrow 2\ Ta_2O_5$
Pr	$4\ Pr + 3\ O_2 \rightarrow 2\ Pr_2O_3$	Cr	$4\ Cr + 3\ O_2 \rightarrow 2\ Cr_2O_3$
Cu	$2\ Cu + O_2 \rightarrow 2\ CuO$	Tb	$4\ Tb + 3\ O_2 \rightarrow 2\ Tb_2O_3$
Bi	$4\ Bi + 3\ O_2 \rightarrow 2\ Bi_2O_3$	Hf	$Hf + O_2 \rightarrow HfO_2$
Ce	$Ce + O_2 \rightarrow CeO_2$	S	$2\ S + 3\ O_2 \rightarrow 2\ SO_3$
Sn	$Sn + O_2 \rightarrow SnO_2$	Zr	$Zr + O_2 \rightarrow ZrO_2$
Ni	$2\ Ni + O_2 \rightarrow 2\ NiO$	Na	$4\ Na + O_2 \rightarrow 2\ Na_2O$
Nb	$4\ Nb + 5\ O_2 \rightarrow 2\ Nb_2O_5$	Ru	$Ru + O_2 \rightarrow RuO_2$
Fe	$4\ Fe + 3\ O_2 \rightarrow 2\ Fe_2O_3$	Ag	$4\ Ag + O_2 \rightarrow 2\ Ag_2O$
K	$4\ K + O_2 \rightarrow 2\ K_2O$	Sr	$2\ Sr + O_2 \rightarrow 2\ SrO$
V	$4\ V + 5\ O_2 \rightarrow 2\ V_2O_5$	Gd	$4\ Gd + 3\ O_2 \rightarrow 2\ Gd_2O_3$
Ho	$4\ Ho + 3\ O_2 \rightarrow 2\ Ho_2O_3$	Lu	$4\ Lu + 3\ O_2 \rightarrow 2\ Lu_2O_3$
Ca	$2\ Ca + O_2 \rightarrow 2\ CaO$	Yb	$4\ Yb + 3\ O_2 \rightarrow 2\ Yb_2O_3$
Mo	$2\ Mo + 3\ O_2 \rightarrow 2\ MoO_3$	Mg	$2\ Mg + O_2 \rightarrow 2\ MgO$
Pt	$Pt + O_2 \rightarrow PtO_2$	Cd	$2\ Cd + O_2 \rightarrow 2\ CdO$
Nd	$4\ Nd + 3\ O_2 \rightarrow 2\ Nd_2O_3$	Y	$4\ Y + 3\ O_2 \rightarrow 2\ Y_2O_3$
Si	$Si + O_2 \rightarrow SiO_2$	La	$4\ La + 3\ O_2 \rightarrow 2\ La_2O_3$
Zn	$2\ Zn + O_2 \rightarrow 2\ ZnO$	Al	$4\ Al + 3\ O_2 \rightarrow 2\ Al_2O_3$
W	$2\ W + 3\ O_2 \rightarrow 2\ WO_3$	Ti	$Ti + O_2 \rightarrow TiO_2$
B	$4\ B + 3\ O_2 \rightarrow 2\ B_2O_3$	As	$4\ As + 3\ O_2 \rightarrow 2\ As_2O_3$
In	$4\ In + 3\ O_2 \rightarrow 2\ In_2O_3$	Ir	$Ir + O_2 \rightarrow IrO_2$
Sb	$4\ Sb + 3\ O_2 \rightarrow 2\ Sb_2O_3$	P	$4\ P + 5\ O_2 \rightarrow 2\ P_2O_5$
Pd	$2\ Pd + O_2 \rightarrow 2\ PdO$	Ge	$Ge + O_2 \rightarrow GeO_2$

atomic_ea_min	atomic_hvap_min	coeff_of_lte_min
atomic_ea_max	atomic_hvap_max	coeff_of_lte_max
atomic_ea_sum	atomic_hvap_sum	coeff_of_lte_sum
atomic_ea_mean	atomic_hvap_mean	coeff_of_lte_mean
atomic_ea_stddev	atomic_hvap_stddev	coeff_of_lte_stddev
atomic_en_allen _min	atomic_radius_min	covalent_rad_min
atomic_en_allen _max	atomic_radius_max	covalent_rad_max
atomic_en_allen _sum	atomic_radius_sum	covalent_rad_sum
atomic_en_allen _mean	atomic_radius_mean	covalent_rad_mean
atomic_en_allen _stddev	atomic_radius_stddev	covalent_rad_stddev
atomic_en_allredroch_min	atomic_radius_calculated_min	covalent_rad_emp_min
atomic_en_allredroch_max	atomic_radius_calculated_max	covalent_rad_emp_max
atomic_en_allredroch_sum	atomic_radius_calculated_sum	covalent_rad_emp_sum
atomic_en_allredroch_mean	atomic_radius_calculated_mean	covalent_rad_emp_mean
atomic_en_allredroch_stddev	atomic_radius_calculated_stddev	covalent_rad_emp_stddev
atomic_en_pauling_min	atomic_spacegroupnum_min	critical_temperature_min
atomic_en_pauling_max	atomic_spacegroupnum_max	critical_temperature_max
atomic_en_pauling_sum	atomic_spacegroupnum_sum	critical_temperature_sum
atomic_en_pauling_mean	atomic_spacegroupnum_mean	critical_temperature_mean
atomic_en_pauling_stddev	atomic_spacegroupnum_stddev	critical_temperature_stddev
atomic_en_sanderson_min	boiling_point_min	density_of_solid_min
atomic_en_sanderson_max	boiling_point_max	density_of_solid_max
atomic_en_sanderson_sum	boiling_point_sum	density_of_solid_sum
atomic_en_sanderson_mean	boiling_point_mean	density_of_solid_mean
atomic_en_sanderson_stddev	boiling_point_stddev	density_of_solid_stddev
atomic_hatm_min	brinell_hardness_min	electrical_resist_min
atomic_hatm_max	brinell_hardness_max	electrical_resist_max
atomic_hatm_sum	brinell_hardness_sum	electrical_resist_sum
atomic_hatm_mean	brinell_hardness_mean	electrical_resist_mean
atomic_hatm_stddev	brinell_hardness_stddev	electrical_resist_stddev
atomic_hfu_min	bulk_mod_min	liquid_range_min
atomic_hfu_max	bulk_mod_max	liquid_range_max
atomic_hfu_sum	bulk_mod_sum	liquid_range_sum
atomic_hfu_mean	bulk_mod_mean	liquid_range_mean
atomic_hfu_stddev	bulk_mod_stddev	liquid_range_stddev
melting_point_min	thermal_conduct_min	atomic_enc_min
melting_point_max	thermal_conduct_max	atomic_enc_max
melting_point_sum	thermal_conduct_sum	atomic_enc_sum
melting_point_mean	thermal_conduct_mean	atomic_enc_mean
melting_point_stddev	thermal_conduct_stddev	atomic_enc_stddev
mineral_hardness_min	supercond_temp_min	atomic_ionization_energy_min

Figure S1: All 246 features and the source of the raw data used for their calculation: blue for NOMAD, green for Mendeleev, and gray for FactSage (page 1 of 2).

mineral_hardness_max	supercond_temp_max	atomic_ionization_energy_max
mineral_hardness_sum	supercond_temp_sum	atomic_ionization_energy_sum
mineral_hardness_mean	supercond_temp_mean	atomic_ionization_energy_mean
mineral_hardness_stddev	supercond_temp_stddev	atomic_ionization_energy_stddev
molar_vol_min	van_der_waals_rad_min	atomic_orbital_radii_min
molar_vol_max	van_der_waals_rad_max	atomic_orbital_radii_max
enthalpy-oxides_min	enthalpy-oxides_max	enthalpy-oxides_sum
refract_index_mean	pauling_ionic_radii_stddev	refract_index_stddev
youngs_mod_sum	electronegativity_ghosh_min	reflectivity_min
Z_min	electronegativity_ghosh_max	poissons_ratio_max
Z_max	electronegativity_ghosh_sum	poissons_ratio_sum
Z_sum	electronegativity_ghosh_mean	poissons_ratio_mean
Z_mean	electronegativity_ghosh_stddev	poissons_ratio_stddev
Z_stddev	VEC_min	van_der_waals_rad_sum
valence_d_electrons_min	VEC_max	van_der_waals_rad_mean
valence_d_electrons_max	VEC_sum	van_der_waals_rad_stddev
valence_d_electrons_sum	VEC_mean	vel_of_sound_min
valence_d_electrons_mean	VEC_stddev	vel_of_sound_max
valence_d_electrons_stddev	pauling_ionic_radii_max	vel_of_sound_sum
vel_of_sound_stddev	pauling_ionic_radii_sum	vel_of_sound_mean
pauling_ionic_radii_mean	gibbs-oxides_min	oxidation_sum
rigidity_mod_min	gibbs-oxides_max	oxidation_mean
rigidity_mod_max	gibbs-oxides_sum	oxidation_min
rigidity_mod_sum	gibbs-oxides_mean	refract_index_max
rigidity_mod_mean	gibbs-oxides_stddev	youngs_mod_max
rigidity_mod_stddev	entropy-oxides_min	molar_vol_sum
atomic_ebe _min	entropy-oxides_max	molar_vol_mean
atomic_ebe _max	entropy-oxides_sum	molar_vol_stddev
atomic_ebe _sum	entropy-oxides_mean	poissons_ratio_min
atomic_ebe _mean	entropy-oxides_stddev	atomic_orbital_radii_sum
atomic_ebe _stddev	deltaCp-oxides_min	atomic_orbital_radii_mean
oxidation_stddev	deltaCp-oxides_max	atomic_orbital_radii_stddev
enthalpy-oxides_mean	deltaCp-oxides_sum	pauling_ionic_radii_min
refract_index_sum	deltaCp-oxides_mean	deltaCp-oxides_stddev
enthalpy-oxides_stddev	entropy_config	vickers_hardness_sum
reflectivity_max	coordination_min	vickers_hardness_mean
reflectivity_sum	coordination_max	vickers_hardness_stddev
reflectivity_mean	coordination_sum	youngs_mod_min
reflectivity_stddev	coordination_mean	coordination_stddev
refract_index_min	vickers_hardness_max	youngs_mod_mean
vickers_hardness_min	oxidation_max	youngs_mod_stddev

Figure S2: All 246 features and the source of the raw data used for their calculation: blue for NOMAD, green for Mendeleev, and gray for FactSage (page 2 of 2).

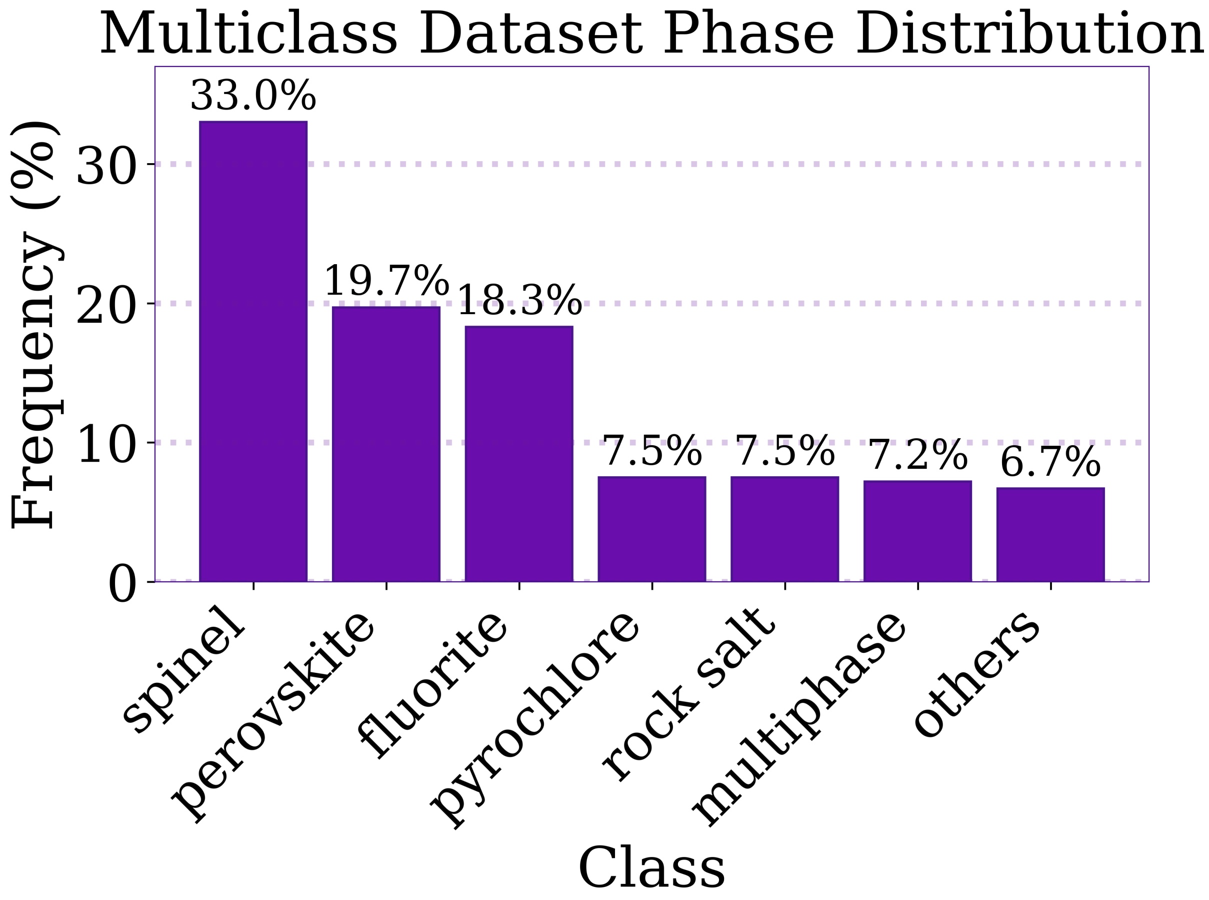

Figure S3: Class distribution in multi-class datasets

Table S2: Selected feature set for 1_ds_Filter_features dataset

**Feature**	**Feature**	**Feature**
atomic_ea_max	atomic_en_allredroch_sum	atomic_radius_max
atomic_radius_calculated_max	bulk_mod_max	covalent_rad_emp_max
covalent_rad_emp_sum	electrical_resist_max	rigidity_mod_max
van_der_waals_rad_mean	entalpia_oxides_min	entropia_oxides_min
Z_sum	—	—

Table S3: Selected features for dataset 2_ds_Filter_features.

No.	Feature
1	atomic_ea_max
2	atomic_en_allredroch_sum
3	atomic_en_sanderson_max
4	atomic_hfu_max
5	atomic_radius_max
6	atomic_radius_calculated_max
7	atomic_radius_calculated_sum
8	atomic_spacegroupnum_mean
9	bulk_mod_max
10	covalent_rad_emp_max
11	covalent_rad_emp_sum
12	density_of_solid_max
13	electrical_resist_max
14	rigidity_mod_max
15	van_der_waals_rad_mean
16	entalpia_oxides_min
17	entropia_oxides_min
18	deltaCp_oxides_max
19	oxidation_sum
20	Z_max
21	Z_sum

Table S4: Selected features for dataset 3_ds_Filter_features.

No.	Feature
1	VEC_cov
2	VEC_min_to_max_ratio
3	Z_cov
4	Z_min_to_max_ratio
5	Z_range
6	atomic_ea_min_to_max_ratio
7	atomic_ea_range
8	youngs_mod_min_to_max_ratio
9	youngs_mod_range
10	atomic_en_allredroch_cov
11	atomic_en_sanderson_cov
12	atomic_en_sanderson_min_to_max_ratio
13	atomic_en_sanderson_range
14	atomic_hfu_min_to_max_ratio
15	atomic_radius_calculated_cov
16	brinell_hardness_range
17	bulk_mod_cov
18	coeff_of_lte_min_to_max_ratio
19	coeff_of_lte_range
20	covalent_rad_emp_cov
21	covalent_rad_emp_range
22	deltaCp_oxides_min_to_max_ratio
23	deltaCp_oxides_range
24	density_of_solid_range
25	electrical_resist_cov
26	electrical_resist_min_to_max_ratio
27	electrical_resist_range
28	entalpia_oxides_cov
29	entalpia_oxides_min_to_max_ratio
30	entalpia_oxides_range
31	mineral_hardness_cov
32	mineral_hardness_min_to_max_ratio
33	mineral_hardness_range
34	oxidation_min_to_max_ratio
35	poissons_ratio_cov
36	reflectivity_cov
37	reflectivity_range
38	rigidity_mod_min_to_max_ratio
39	rigidity_mod_range
40	supercond_temp_cov
41	supercond_temp_range
42	thermal_conduct_min_to_max_ratio
43	thermal_conduct_range
44	valence_d_eletrons_cov

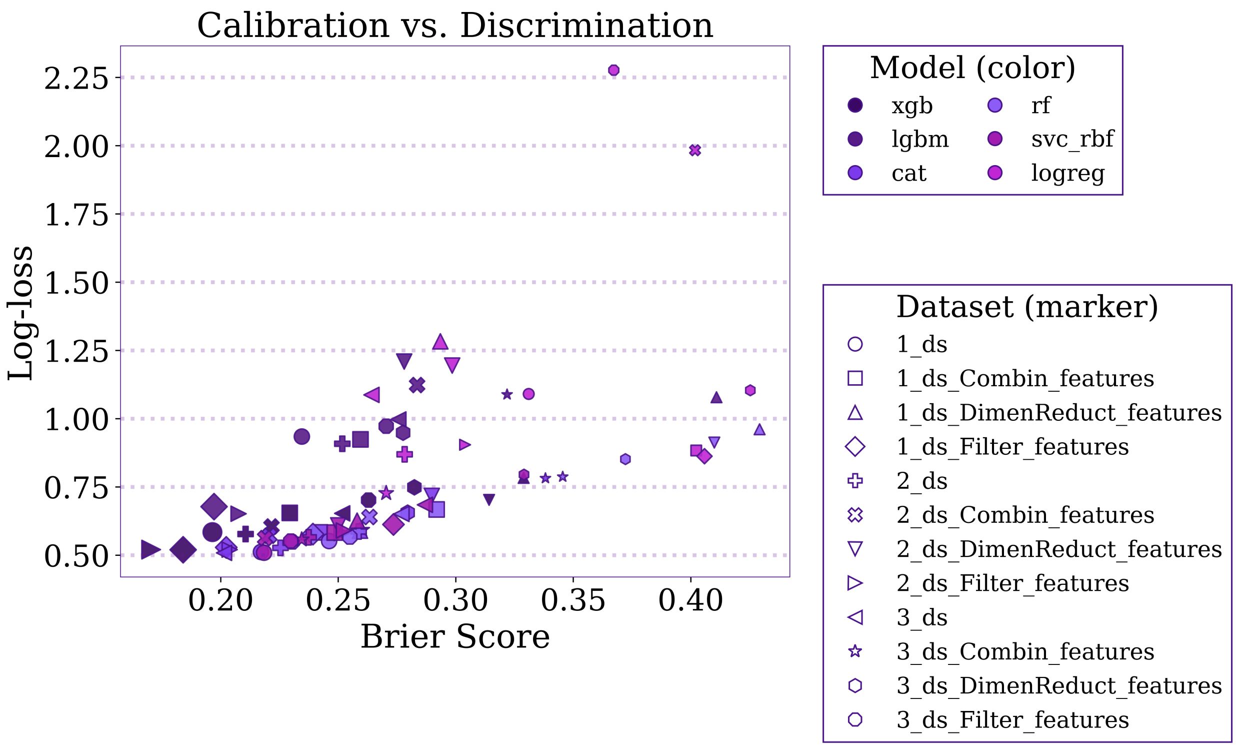

Figure S4: Brier Score and Log Loss Analysis of calibration–discrimination trade-off in multi-class classification

Table S5: Best hyperparameters obtained for the MLP network.

Hyperparameter	Best value
`n_layers`	1
`n_units_l1`	24
`activation`	`tanh`
`solver`	`adam`
`alpha`	2.34×10^{-3}
`tol`	1.23×10^{-5}
`max_iter`	30000
`shuffle`	`True`
`warm_start`	`False`
`batch_choice`	`auto`
`learning_rate_init`	1.14×10^{-3}
`beta_1`	0.8891631637
`beta_2`	0.9995138880
`epsilon`	1.61×10^{-10}

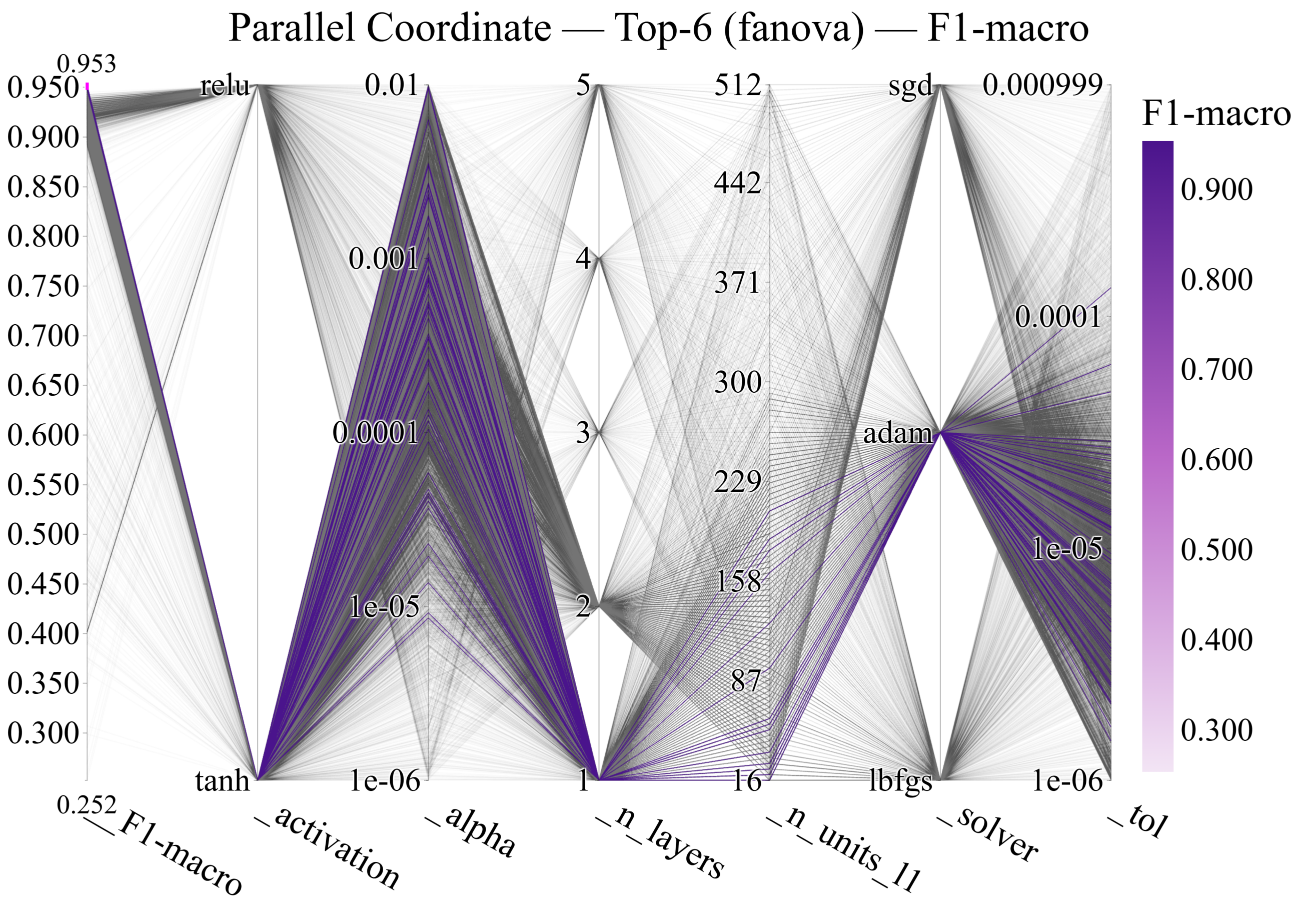

Figure S5: Parallel Coordinates plots. (MLP)

Table S6: Distribution of phases in the dataset for binary classification.

Phase	Count	Percentage (%)
`other`	477	67.09
`perovskite`	234	32.91
Total	711	100.00

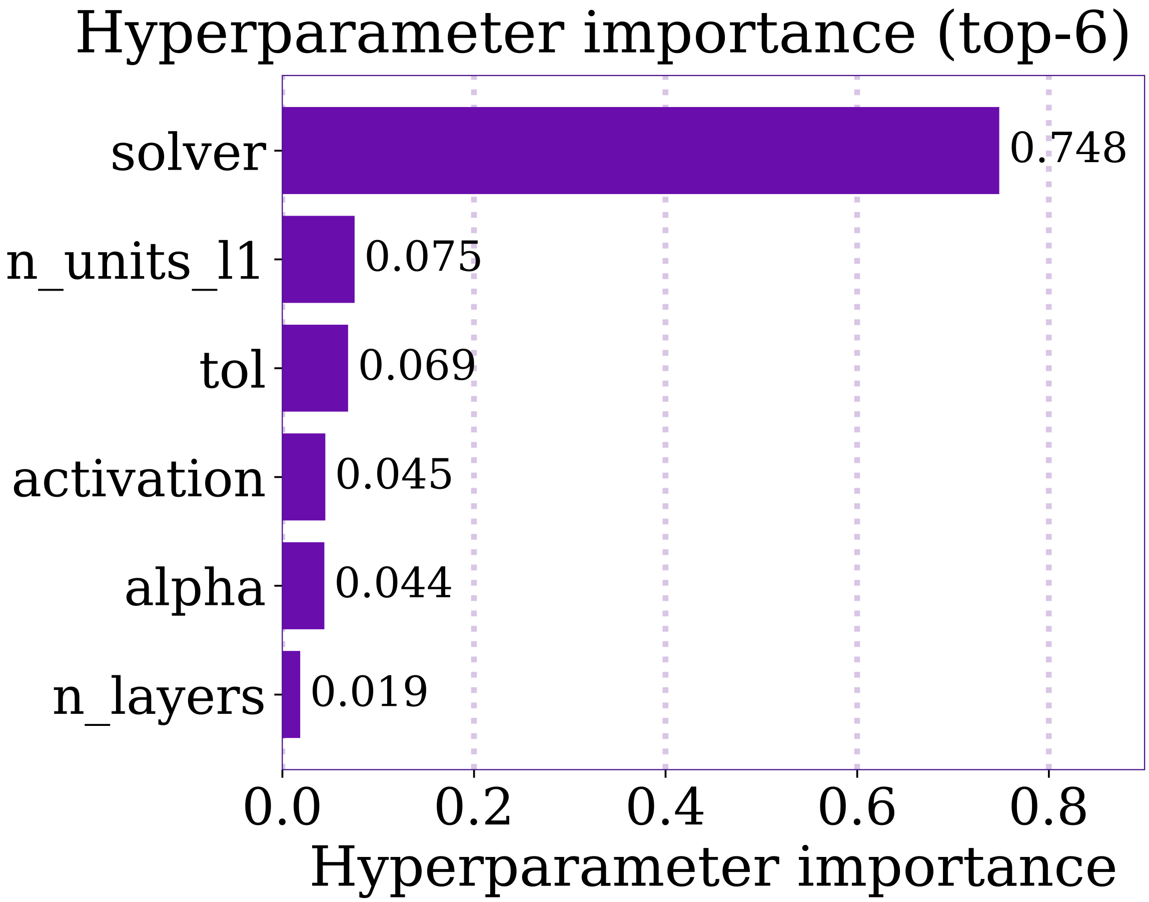

Figure S6: Hyperparameter importance bar plot. (MLP)

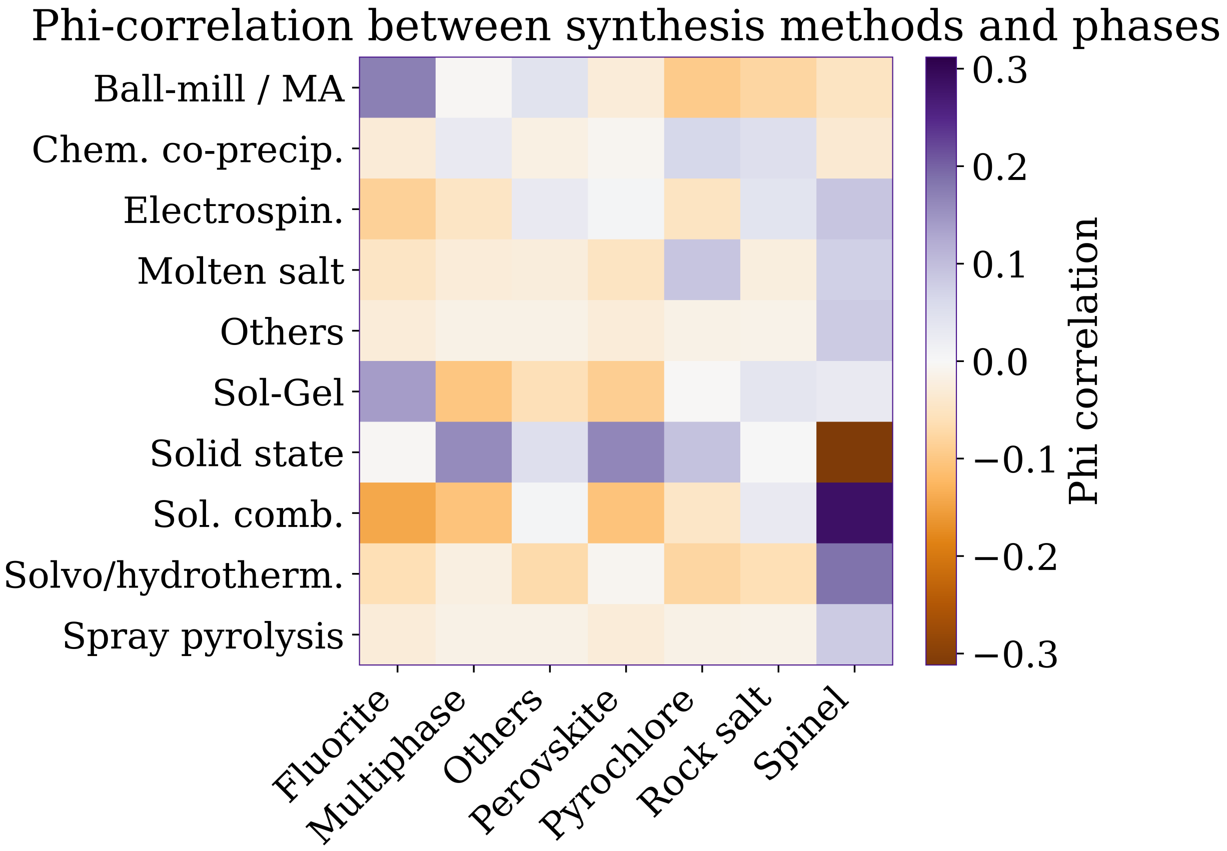

Figure S7: ϕ-correlation between synthesis methods and crystallographic phases. Abbreviated synthesis labels on the y-axis are: *Ball-mill / MA* (ball-mill / mechanical alloying / mechanochemistry), *Chem. co-precip.* (chemical co-precipitation), *Electrospin.* (electrospinning), *Molten salt* (molten salt synthesis), *Sol-Gel* (sol–gel), *Solid state* (solid-state reaction), *Sol. comb.* (solution combustion), *Solvo/hydrotherm.* (solvothermal / hydrothermal), *Spray pyrolysis* (spray-pyrolysis method), and *Others* (all remaining synthesis routes).

Table S7: Selected features for MLP by SHAP.

ID	Feature	ID	Feature
1	critical_temperature_max	2	electrical_resist_sum
3	critical_temperature_mean	4	critical_temperature_sum
5	critical_temperature_stdv	6	electrical_resist_stdv
7	electrical_resist_mean	8	vickers_hardness_min
9	electrical_resist_max	10	atomic_en_sanderson_min_to_max_ratio
11	electrical_resist_range	12	atomic_hfu_std_over_range
13	gibbs-oxides_min_to_max_ratio	14	atomic_ea_min_to_max_ratio
15	rigidity_mod_min_to_max_ratio	16	deltacp-oxides_min_to_max_ratio
17	deltacp-oxides_cov	18	reflectivity_min
19	covalent_rad_emp_sum	20	poissons_ratio_min_to_max_ratio
21	enthalpy-oxides_max	22	thermal_conduct_min
23	covalent_rad_range	24	deltacp-oxides_min
25	atomic_ea_std_over_range	26	bulk_mod_min_to_max_ratio
27	gibbs-oxides_max	28	entropy-oxides_mean
29	youngs_mod_sum	30	covalent_rad_min_to_max_ratio
31	coeff_of_lte_std_over_range	32	youngs_mod_cov
33	valence_d_electrons_cov	34	youngs_mod_min
35	vec_stdv	36	atomic_en_sanderson_min
37	enthalpy-oxides_std_over_range	38	refract_index_stdv
39	covalent_rad_emp_stdv	40	oxidation_std_over_range
41	rigidity_mod_std_over_range	42	coeff_of_lte_min
43	vec_sum	44	covalent_rad_stdv
45	deltacp-oxides_range	46	z_std_over_range
47	atomic_spacegroupnum_std_over_range	48	rigidity_mod_range
49	deltacp-oxides_std_over_range	50	boiling_point_stdv
51	atomic_en_allen__max	52	atomic_hatm_mean
53	atomic_en_sanderson_max	54	atomic_en_allredroch_max
55	youngs_mod_range		

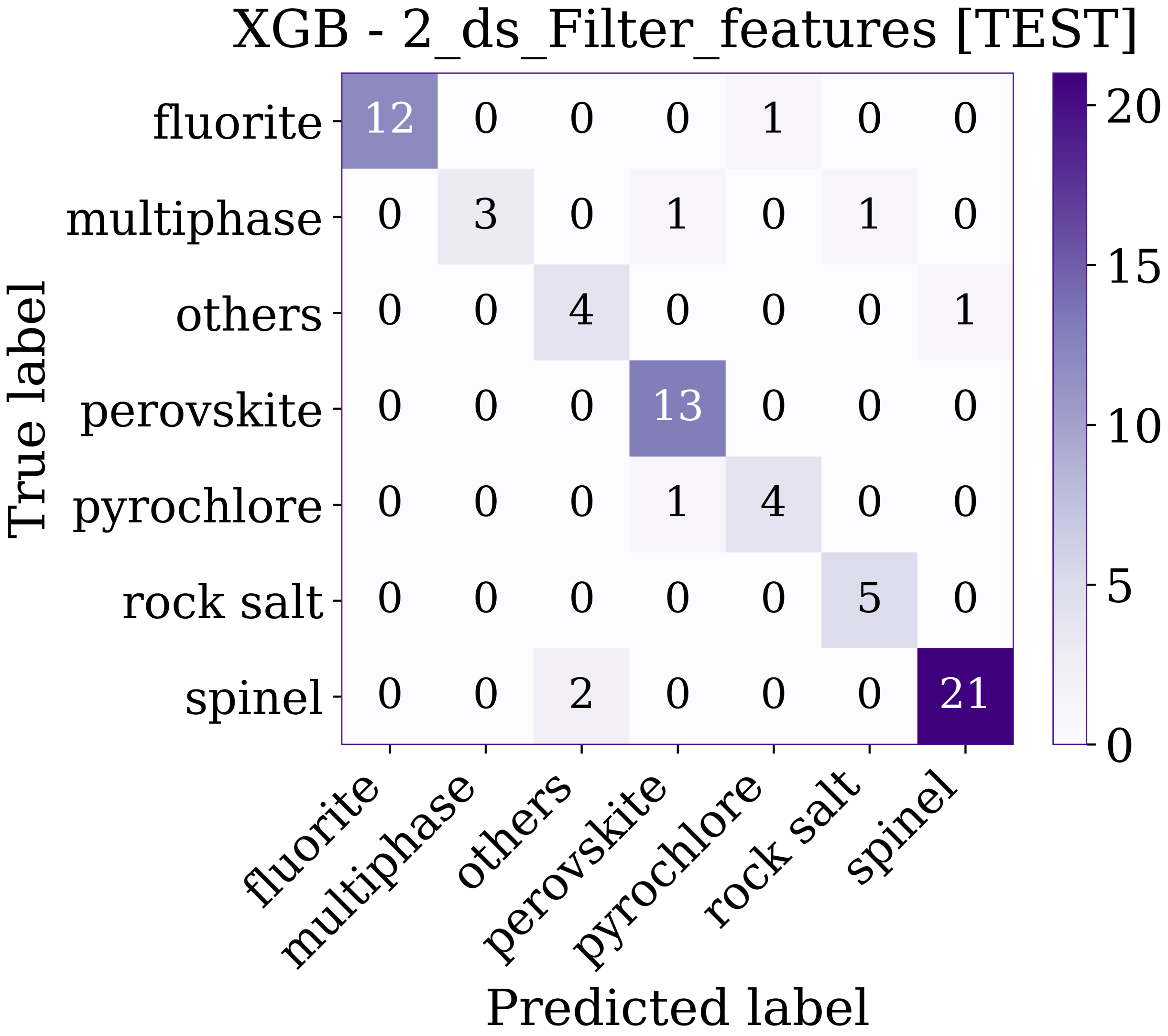

Figure S8: Confusion Matrix Visualization of XGBoost Model Performance on 2_ds_Filter_features Dataset (Test Set)

Box S1. Prompt designed for extracting HEO data from scientific abstracts.

ROLE You are an information extractor for scientific abstracts about high-entropy oxides (HEOs).

OBJECTIVE From a single abstract, return a JSON object containing the abstract text and a list of per-composition records. Each record must include: composition, whether it qualifies as an HEO (oxygen present + ≥ 5 distinct cations), the phase, and any synthesis / sintering / deposition methods that are explicitly linked to that composition in the abstract.

STRICT OUTPUT CONTRACT Return exactly one JSON object with these keys and rules:

"abstract": "<the input abstract verbatim>", "items": ["composition": "<normalized chemical formula>", "is_high_entropy_oxide": true, "cation_count": 0, "has_oxygen": true, "phase": "<one of the allowed_phase_labels or null>", "phase_evidence": "<exact string span quoted from the abstract or null>", "synthesis_method": "<one of the allowed_synthesis_labels or null>", "synthesis_evidence": "<exact string span or null>", "sintering_method": "<one of the allowed_sintering_labels or null>", "sintering_evidence": "<exact string span or null>", "deposition_method": "<one of the allowed_deposition_labels or null>", "deposition_evidence": "<exact string span or null>", "variables_expanded_from": "<e.g., 'M=Cu,Mg,Zn' if applicable, else null>", "notes": "<brief 1-line caution if anything ambiguous, else ''>"], "warnings": ["<schema or qualification issues detected; empty if none>"]

Never invent values. If an item is not present verbatim in the abstract, return null for that field.

Evidence fields must be exact substrings quoted from the abstract (no paraphrases).

If multiple compositions are mentioned, create one items[] entry per composition.

If the abstract uses variables like M or X (e.g., La(Cr0.2Fe0.2Mn0.2Ni0.2M0.2)O-

3 (M=Cu, Mg, Zn)), enumerate all concrete compositions and list each as a separate items[] entry. Populate variables_expanded_from with the mapping (e.g., "M=Cu,Mg,Zn").

QUALIFICATION RULE (HEO gate) A composition counts as a high-entropy oxide only if:

It contains oxygen (O), and

It contains ≥ 5 distinct cations (element symbols with positive oxidation states; treat H, C, N as cations only if explicitly used as cations in oxides, which is rare).

Populate is_high_entropy_oxide, cation_count, and has_oxygen accordingly. If either condition fails, exclude the composition from items unless the abstract explicitly calls it "high-entropy oxide"; in that outlier case, include it but set a notes warning like: "marked as HEO by authors but <reason>".

NORMALIZATION RULES

Normalize hyphenation artifacts: convert O-3 → O3, while leaving units text unchanged.

Preserve element capitalization and decimal subscripts: La(Cr0.2Fe0.2...)O3.

Accept both rock salt and rocksalt → canonical label: rock salt.

If the abstract states multiple stable phases for the same composition under ambient conditions, label phase = "multiphase". If phase changes are reported only under irradiation/charging/etc., do not label as multiphase; prefer the ambient/stable phase. If unclear, use null and note why in notes.

ALLOWED LABELS Use only these canonical labels (verbatim) or null:

allowed_phase_labels: ["perovskite","spinel","monoclinic","pyrochlore", "fluorite","rock salt","cubic perovskite","orthorhombic perovskite","double perovskite","quadruple perovskite","tetragonal perovskite","bixbyite","magnetoplumbite","O3-type layered","Layered Ruddlesden–Popper","hexaaluminates","mullite","olivine",

"monosilicates","disilicates","aluminates","amorphous","multiphase"]
allowed_synthesis_labels: ["Solid state reaction","Solution combustion","Sol-Gel","Solvo/hydrothermal","Chemical co-precipitation","Ball-mill / mechanical alloying / mechanochemistry","Electrospinning","Spray pyrolysis method","Molten salt synthesis"]
allowed_sintering_labels: ["Solid-state sintering","Spark plasma sintering","Flash sintering","Microwave sintering","Ultrafast high-temperature sintering"]
allowed_deposition_labels: ["Magnetron sputtering deposition","Chemical Vapor Deposition","Physical Vapor Deposition"]
SYNONYM → CANONICAL MAPPING (apply before output)
Phases: rocksalt→rock salt; M-type hexaferrite→magnetoplumbite; Ruddlesden–Popper / RP→Layered Ruddlesden–Popper.
Synthesis: glycine-nitrate process (GNP)→Solution combustion; co-precipitation→Chemical co-precipitation; mechanochemistry / high-energy ball milling→Ball-mill / mechanical alloying / mechanochemistry; hydrothermal / solvothermal→Solvo/hydrothermal.
Sintering: SPS→Spark plasma sintering; UHS→Ultrafast high-temperature sintering.
Deposition: magnetron sputtering→Magnetron sputtering deposition; CVD→Chemical Vapor Deposition; PVD→Physical Vapor Deposition.
ASSIGNMENT RULES
Linkage discipline: Only assign a phase or method to a composition if the abstract explicitly ties them. If the abstract states a method globally (“powders were synthesized by…”), you may apply it to all listed compositions only if they are presented as part of the same synthesis batch/series. Otherwise, leave null.
Evidence fields must quote the exact text that justifies the label (e.g., "single-phase perovskite structure"; "synthesized by solution combustion").

If information is missing or ambiguous, return null for that field and add a short notes clarification.

DETAILED EXAMPLE

Example input (abstract): "High-entropy oxides (HEOs) were explored to tailor structure and transport. A series La(Cr0.2Fe0.2Mn0.2Ni0.2M0.2)O-3 (M = Cu, Mg, Zn) were synthesized by solution combustion via the glycine-nitrate process (GNP); the resulting powders were spark plasma sintered (SPS) at 1100 °C for 5 min. Room-temperature XRD indicates a single-phase perovskite structure for all compositions; under in-situ irradiation the La(Cr0.2Fe0.2Mn0.2Ni0.2Zn0.2)O-3 shows a transient orthorhombic distortion that reverts after cooling. Additionally, rocksalt-type (Co0.2Ni0.2Mg0.2Zn0.2Cu0.2)O was prepared by chemical co-precipitation followed by solid-state sintering; XRD reports rocksalt structure. A dual-phase spinel + fluorite at room temperature was observed in a composition denoted (Fe0.2Co0.2Ni0.2Mg0.2Cu0.2)O_x after molten salt synthesis. Thin films of LaNiO3 were deposited by magnetron sputtering; although described as a high-entropy oxide thin film, LaNiO3 contains oxygen and only two distinct cations."

Corresponding output (JSON):

"abstract": "High-entropy oxides (HEOs) were explored to tailor structure and transport. A series La(Cr0.2Fe0.2Mn0.2Ni0.2M0.2)O-3 (M = Cu, Mg, Zn) were synthesized by solution combustion via the glycine-nitrate process (GNP); the resulting powders were spark plasma sintered (SPS) at 1100 °C for 5 min. Room-temperature XRD indicates a single-phase perovskite structure for all compositions; under in-situ irradiation the La(Cr0.2Fe0.2Mn0.2Ni0.2Zn0.2)O-3 shows a transient orthorhombic distortion that reverts after cooling. Additionally, rocksalt-type (Co0.2Ni0.2Mg0.2Zn0.2Cu0.2)O was prepared by chemical co-precipitation followed by solid-state sintering; XRD reports rocksalt structure. A dual-phase spinel + fluorite at room temperature was ob-

served in a composition denoted (Fe0.2Co0.2Ni0.2Mg0.2Cu0.2)O_x after molten salt synthesis. Thin films of LaNiO3 were deposited by magnetron sputtering; although described as a high-entropy oxide thin film, LaNiO3 contains oxygen and only two distinct cations.", "items": ["composition": "La(Cr0.2Fe0.2Mn0.2Ni0.2Cu0.2)O3", "is_high_entropy_oxide": true, "cation_count": 6, "has_oxygen": true, "phase": "perovskite", "phase_evidence": "single-phase perovskite structure", "synthesis_method": "Solution combustion", "synthesis_evidence": "synthesized by solution combustion via the glycine-nitrate process (GNP)", "sintering_method": "Spark plasma sintering", "sintering_evidence": "spark plasma sintered (SPS) at 1100 °C for 5 min", "deposition_method": null, "deposition_evidence": null, "variables_expanded_from": "M=Cu,Mg,Zn", "notes": "" , "composition": "La(Cr0.2Fe0.2Mn0.2Ni0.2Mg0.2)O3", "is_high_entropy_oxide": true, "cation_count": 6, "has_oxygen": true, "phase": "perovskite", "phase_evidence": "single-phase perovskite structure", "synthesis_method": "Solution combustion", "synthesis_evidence": "synthesized by solution combustion via the glycine-nitrate process (GNP)", "sintering_method": "Spark plasma sintering", "sintering_evidence": "spark plasma sintered (SPS) at 1100 °C for 5 min", "deposition_method": null, "deposition_evidence": null, "variables_expanded_from": "M=Cu,Mg,Zn", "notes": "" , "composition": "La(Cr0.2Fe0.2Mn0.2Ni0.2Zn0.2)O3", "is_high_entropy_oxide": true, "cation_count": 6, "has_oxygen": true, "phase": "perovskite", "phase_evidence": "single-phase perovskite structure", "synthesis_method": "Solution combustion", "synthesis_evidence": "synthesized by solution combustion via the glycine-nitrate process (GNP)", "sintering_method": "Spark plasma sintering", "sintering_evidence": "spark plasma sintered (SPS) at 1100 °C for 5 min", "deposition_method": null, "deposition_evidence": null, "variables_expanded_from": "M=Cu,Mg,Zn", "notes": "Transient orthorhombic distortion under in-situ irradiation is not the ambient phase."

, "composition": "Co0.2Ni0.2Mg0.2Zn0.2Cu0.2O", "is_high_entropy_oxide": true, "cation_count": 5, "has_oxygen": true, "phase": "rock salt", "phase_evidence": "rocksalt structure", "synthesis_method": "Chemical co-precipitation", "synthesis_evidence": "chemical co-precipitation", "sintering_method": "Solid-state sintering", "sintering_evidence": "solid-state sintering", "deposition_method": null, "deposition_evidence": null, "variables_expanded_from": null, "notes": "" , "composition": "(Fe0.2Co0.2Ni0.2Mg0.2Cu0.2)O_x", "is_high_entropy_oxide": true, "cation_count": 5, "has_oxygen": true, "phase": "multiphase", "phase_evidence": "dual-phase spinel + fluorite at room temperature", "synthesis_method": "Molten salt synthesis", "synthesis_evidence": "molten salt synthesis", "sintering_method": null, "sintering_evidence": null, "deposition_method": null, "deposition_evidence": null, "variables_expanded_from": null, "notes": "" , "composition": "LaNiO3", "is_high_entropy_oxide": false, "cation_count": 2, "has_oxygen": true, "phase": null, "phase_evidence": null, "synthesis_method": null, "synthesis_evidence": null, "sintering_method": null, "sintering_evidence": null, "deposition_method": "Magnetron sputtering deposition", "deposition_evidence": "Thin films of LaNiO3 were deposited by magnetron sputtering", "variables_expanded_from": null, "notes": "marked as HEO by authors but cation_count=2 < 5 (see: high-entropy oxide thin film)"], "warnings": ["Included a non-HEO item because authors explicitly labeled it 'high-entropy oxide thin film'; verify downstream filters if strict HEO-only output is required."]

Box S2. Fraction calculation instructions.

You will receive a chemical composition written in chemical notation and the phase in which the system is found. The phase and the use of parentheses in the formulas provide relevant information about the occupancy and division of cations within the same site. Your task is to calculate the relative proportion of each atom, disregarding oxygen.

You must generate a JSON as the output, following this example: "Normalized-proportions": ["x.xxxx","y.yyyy", ...] This is a list of relative proportions of cations, where the numbers are decimals with exactly four decimal places, and the total sum of the list is equal to one (normalized list).
For a correct calculation, it is crucial that you interpret the occupancy and division of cations according to the sites indicated in the phase, or by analyzing the chemical formula, which may sometimes separate sites using parentheses and/or brackets. Additionally, multipliers may appear after parentheses, altering the fractions of the internal elements. Therefore, you must read the chemical expression according to the conventions of chemical composition writing, integrate your considerations of the given phase, and only then perform the proportion calculations.
Before giving the final answer, perform a sanity check. Verify that you correctly interpreted the mentioned phase and the chemical formula notation with parentheses and brackets. In other words, check whether the sum of the atomic fractions matches what is expected for each site present in the system. Remember that these are high-entropy oxides, so in some cases, such as certain spinels, atoms may be mixed between the A and B sites, meaning there may be only one site if parentheses are not specified. On the other hand, if parentheses are written, this indicates that there are two sites in the structure. Pay close attention to the correct interpretation of the sites and the atomic distribution of the elements within these sites.
All compositions are high-entropy oxide compositions. Your output must be only the JSON, with no additional comments.
Example input: (La0.5Gd0.5Ce0.5Y0.5)2Zr2O7 pyrochlore Interpretation: In the input formula, it is possible to observe parentheses. Since this is a pyrochlore structure, these parentheses can be interpreted as the separation of an A-site and a B-site. As the structure of pyrochlore is $A_2B_2O_7$, we can immediately see that Zr is 0.5000, because

it is the only cation occupying the B-site. In the A-site, the cations together complete the other 0.5000, since the indices of the A and B sites are equal. Therefore, La, Gd, Ce, and Y = 0.1250 each. Example output: ["0.1250", "0.1250", "0.1250", "0.1250", "0.5000"]

Example input: `Sr(CrMnFeCoNi)(12)O19` multiphase

Interpretation: I treated it as explicit cation counting: Sr = 1; `(CrMnFeCoNi)(12)` → 12 atoms split equally (2.4 each); total cations = 13; I normalized by 13 to obtain the fractions (Sr = 1/13 ≈ 0.0769; each of Cr/Mn/Fe/Co/Ni = 2.4/13 ≈ 0.1846).

Example output: `["0.0769","0.1846","0.1846","0.1846","0.1846","0.1846"]`

Example input: `[CaNaPbSr(LaNdPr)]TiO`$_3$ tetragonal perovskite

Interpretation: I treat it as an ABO_3 perovskite with implicit indices: `[CaNaPbSr(LaNdPr)]` fills the A site (= 1); assign one share to each species outside the parentheses (Ca, Na, Pb, Sr) and one share to the `(LaNdPr)` group; normalize 5 shares → 0.8 for the Ca/Na/Pb/Sr block and 0.2 for `(LaNdPr)`; divide equally within each subset (Ca, Na, Pb, Sr = 0.20 each; La, Nd, Pr ≈ 0.0667 each); Ti = 1 at the B site; if fractions over the total cations are desired, renormalize by $(A + B) = 2$.

Example output:

```
["0.1000","0.1000","0.1000","0.1000",
"0.0333","0.0333","0.0333","0.5000"]
```

Example input: [Li0.15(BaSrCaMg)0.2125]TiO3 cubic perovskite Interpretation: I treat it as an ABO_3 perovskite with explicit indices on the A site: in [Li0.15(BaSrCaMg)0.2125], Li = 0.15 and 0.2125 multiplies Ba, Sr, Ca, and Mg (the A site already sums to 1, so no quota-style normalization); Ti = 1 on the B site; if fractions over the total cations are desired, renormalize by (A+B) = 2. Example output: ["0.0750", "0.1062", "0.1062", "0.1062", "0.1062", "0.5000"]

```
{
    "$schema": "https://json-schema.org/draft/2020-12/schema",
    "title": "MIDB NLPE json schema",
    "description": "Schema for NLPE dataset validation on MIDB",
    "type": "object",
    "properties": {
        "application": { "type": "string", "enum": ["NLP-Extraction"]},
        "version": { "type": "string", "pattern": "^[1-9].[0-9].[0-9]$"},
        "extraction": {
            "type": "object",
            "properties": {
                "target": {
                    "type": "array",
                    "items": {"type": "string", "minItems": 1}
                },
                "method": {
                "type": "string",
                    "enum": ["large language model", "regex", "manual"],
                },
                "model": { "type": "string" },
                "postprocessed": { "type": "boolean" },
                "required": ["target"]
            },
            "date": {
                "type": "string",
                "pattern": "[0-9]{4}-(0[1-9]|1[0-2])-(0[1-9]|1[0-9]|2[0-9]|3[0-1])"
                "T(0[0-9]|1[0-9]|2[0-3]):([0-5][0-9])(:([0-5][0-9]))?"
                "((\\+|-)?(((0[0-9]|1[0-2]):([0-9][0-5])))|([A-Za-z]+))?$"
            },
            "authors": {
                "type": "array",
                "items": {"type": "string", "minItems": 1}
            },
            "extractions": {
                "type": "array",
                "items": {
                    "type": "object",
                    "properties": {
                        "source_text": {"type": "string"},
                        "composition": {"type": "string"},
                        "elements": {
                            "type": "array",
                            "items": {"type": "string"}
                        },
                        "fractions": {
                            "type": "array",
                            "items": {"type": "number"}
                        },
                        "phase": {"type": "string"},
                        "source_doi": {"type": "string"}
                    },
                    "required": ["source_text", "source_doi"]
                }
            }
        }
    },
    "required": ["extractions", "authors", "date", "extraction", "version", "application"]
}
```

Figure S9: json-schema for *.nlpe file validation.
